# Standardising evidence strength grading for recommendations from multiple clinical practice guidelines: a South African case study

**DOI:** 10.1186/s13012-018-0803-0

**Published:** 2018-08-29

**Authors:** K. Grimmer, Q. Louw, J. M. Dizon, S-M van Niekerk, D. Ernstzen, C. Wiysonge

**Affiliations:** 1 Clinical Teaching and Education Centre, College of Nursing and Health Sciences, Flinders University, Daw Park, Adelaide, South Australia 5041 Australia; 2 Department of Physiotherapy, Faculty of Medicine and Health Sciences, Stellenbosch University, Francie van Zijl Drive, Tygerberg, Cape Town, 7505 South Africa; 3 International Centre for Allied Health Evidence, University of South Australia, City East Campus, Adelaide, 5000 Australia; 4 Cochrane South Africa, South African Medical Research Council, Francie van Zijl Drive, Parow Valley, Cape Town, 7505 South Africa

**Keywords:** Clinical practice guidelines, Guidance documents, Methods, Allied health stroke rehabilitation, Resource poor environments, South Africa

## Abstract

**Background:**

Significant resources are required to write de novo clinical practice guidelines (CPGs). There are many freely-available CPGs internationally, for many health conditions. Developing countries rarely have the resources for de novo CPGs, and there could be efficiencies in using CPGs developed elsewhere. This paper outlines a novel process developed and tested in a resource-constrained country (South Africa) to synthesise findings from multiple international CPGs on allied health (AH) stroke rehabilitation.

**Methods:**

Methodologists, policy-makers, content experts and consumers collaborated to describe the pathway of an ‘average’ stroke patient through the South African public healthcare system and pose questions about best-practice stroke rehabilitation along this pathway. A comprehensive search identified international guidance documents published since January 2010. These were scanned for relevance to the South African AH stroke rehabilitation questions and critically appraised for methodological quality. Recommendations were extracted from guidance documents for each question. Strength of the body of evidence (SoBE) gradings underpinning recommendations were standardised, and composite recommendations were developed using qualitative synthesis. An algorithm was developed to guide assignment of overall SoBE gradings to composite recommendations.

**Results:**

Sixteen CPGs were identified, and all were included, as they answered different project questions differently. Methodological quality varied and was unrelated to currency. Seven clusters, outlining 20 composite recommendations were proposed (organise for best practice rehabilitation, operationalise strategies for best practice communication throughout the patient journey, admit to an acute hospital, refer to inpatient rehabilitation, action inpatient rehabilitation, discharge from inpatient rehabilitation and longer-term community-based rehabilitation).

**Conclusion:**

The methodological development process, tested by writing a South African AH stroke rehabilitation guideline from existing evidence sources, took 9 months. The process was efficient, collaborative, effective, rewarding and positive. Using the proposed methods, similar synthesis of existing evidence could be conducted in shorter time periods, in other resource-constrained countries, avoiding the need for expensive and time-consuming de novo CPG development.

**Electronic supplementary material:**

The online version of this article (10.1186/s13012-018-0803-0) contains supplementary material, which is available to authorized users.

## Background

Over the past decade, the South African burden of disease has swung towards chronic conditions, trauma and disability, as more lives are saved from communicable diseases [1, 2]. The shift from communicable disease mortality, to communicable and non-communicable disease morbidity, puts the spotlight firmly on the need for evidence-based rehabilitation, to ensure that resources are wisely allocated to achieve best health and cost outcomes for people living with sequelae of illness and injury [3]. South Africa (SA) has been described as an anomaly among developing countries, having features of high-income country economies (such as good infrastructure) but also features of low-middle-income country economies, with its social and economic problems and continuing need for development aid [4].

Effectively implementing evidence-based practice, particularly using comprehensive high quality clinical practice guidelines (CPGs), has been the subject of considerable research in high-income countries over the past three decades [5]. Much of this research has focused on why so much difficulty is experienced by policy-makers, managers and clinicians in implementing evidence into practice [6]. Across health disciplines, there are generally positive attitudes to using evidence in practice, and despite discipline-differences in competencies and scope of practice, similar reasons emerge for not actually doing so [7, 8]. Commonly, these are lack of time, ready access to CPGs, understanding about different forms of evidence recommendations (e.g. CPGs, protocols, guidance) and knowledge about evaluating evidence quality. Barriers also include disagreement with recommendations, unwillingness to change practices, peer-pressure, lack of managerial and organisational support and differences between recommendations and clinical realities [7–9].

Research into evidence implementation and uptake in low and lower-middle-income countries has largely been in knowledge translation into policy, which has concurrently identified gaps between research and end-user/stakeholder needs for guidance [10]. The challenges of evidence implementation into clinical practice in these countries are yet to be fully identified or addressed [11].

This paper outlines a novel process of using existing best evidence sources to develop new, composite, implementation-ready, evidence-based recommendations. The process was tested on allied health (AH) rehabilitation for a high-burden health condition in a resource-constrained environment (stroke). AH disciplines such as physiotherapy, occupational therapy, speech and language therapy, clinical nutrition and social work provide rehabilitation services to stroke survivors, with the aim of optimising function and quality of life. A 2015 summary of systematic reviews of the impact of AH care on stroke rehabilitation outcomes showed significant economic benefits [12]. Key barriers to implementing evidence-based AH rehabilitation in SA are similar to those reported in high-income countries (for instance lack of workforce, training, support, resources and recognition of effort) [8, 9, 13]. However, there are also unique SA-context facilitators which mediate some of these barriers to improve AH rehabilitation, including the innovative use of resources and informal AH rehabilitation networks with the common goal of improving functioning and quality of life [13].

The World Health Organization (WHO) notes that AH rehabilitation services are generally poorly accessed and/or suboptimal in many low and low-middle economy countries [3]. The World Health Assembly resolution on disability, including prevention, management and rehabilitation, identified that effective AH rehabilitation could contribute to reducing poverty through improving functioning, activity levels and participation. Inefficient and ineffective rehabilitation can cause health deterioration, which is associated with an increased rate of complications and healthcare utilisation [3].

Stroke is a leading cause of disability worldwide [14]. Over the past 40 years, stroke rates in poor economies such as southern India and rural SA have approximately doubled, whereas rates in upper-middle- and high-income economy countries have decreased. The most striking problem is that disability- and mortality-rates from stroke are at least tenfold greater in medically under-served countries versus medically well-served countries [14]. The causes of these disparities are explained by reduced access to services such as early stroke screening, effective early medical management, post-stroke rehabilitation and secondary stroke prevention. The WHO promotes evidence-based public health programs for stroke prevention, management and rehabilitation worldwide, however, the success of such programs depends on government commitment to evidence availability, uptake and implementation [3].

In SA, stroke is a high health burden [1, 2]. It is estimated that 240 people have a stroke each day, which translates into ten strokes each hour [15]. Stroke now affects many young South Africans in their 20s and 30s due to co-morbidities such as HIV/AIDS. In SA, stroke is a leading cause of disability among adults of all ages [15], contributing significantly to healthcare-costs with long-term implications, particularly if rehabilitation is sub-optimal.

South African AH stroke rehabilitation services are currently not supported by local CPGs that summarise the AH literature [16]. Moreover, there is no nationally-recommended CPG from another country that is routinely used by South African AH stroke rehabilitation providers. The lack of local guidance perhaps underpins variable stroke-rehabilitation outcomes across SA [2]. This concurs with the WHO report, which suggests that only 26–55% of people in poorer countries receive the rehabilitation services they need [3]. The World Health Survey analysis indicates that people with disabilities were more than twice as likely to find healthcare provider skills or equipment inadequate and nearly three times more likely to be denied care [3]. South African AH providers require access to good quality, locally-relevant guidance to support them to deliver best-practice stroke rehabilitation to the growing number of South Africans who require it [1, 2]. They also require a process by which guidance can be readily updated to ensure that the practice remains current.

The aims of the research were to develop a template for producing defensible, locally-implementable recommendations from existing evidence sources, and test this by writing an implementation-ready SA-contextualised AH stroke rehabilitation guideline relevant to any South African public healthcare setting.

## Methods

### Reporting standard

The RIGHT Statement was applied [17] (See Additional file 1).

### Ethics approval

Stellenbosch University Africa Human Research Ethics Committee (#0642).

### Funding

Seed funding was provided from Stellenbosch University and the WHO Alliance for Health Policy and Systems Research in February 2017. The funders had no influence on research design, conduct or reporting.

### Project team

This comprised 15 expert clinicians, academics, policy-makers and consumer representatives and five researchers. Other than the consumer representative (a stroke survivor), each team member had an AH discipline background. Each team member brought different understandings of stroke rehabilitation, such as how, when and why stroke rehabilitation is offered, what care is provided, who provides it, how it is received and in what rehabilitation settings. Project funding was sufficient for travel and meeting costs and to support one researcher 2 days/week. All other team members participated voluntarily or as part of usual work commitments.

### Project framework

The timeframe was short (9 months) (Feb–Nov 2017), reflecting the urgent need for effective, efficient, equitable and safe AH rehabilitation for patients with acute and chronic stroke across South African healthcare settings. There was no time or funding to develop a de novo CPG for South African AH stroke rehabilitation, which outlined ‘what’ to do. The focus needed to be on implementing best evidence into local practice, by addressing service delivery questions of ‘who’, ‘how’, ‘when’, ‘where’, ‘why’ and ‘how much’. The project team agreed that existing CPGs would form the evidence base.

The research adopted the WHO characteristics for good quality service delivery [18]. This separates current best-practice information (largely derived from non-biased comparative or intervention studies) from service operationalisation issues. These speak to inputs such as workforce, service comprehensiveness, resources, continuity, coordination, accountability and outputs (quality care processes and health outcomes). Outputs can be variably measured by person-centredness, efficiency, equality (individual rights to care), equity (coverage), access, timeliness and effectiveness.

### Terminology

Any document published from 2010, which provided freely- and publicly-available guidance to inform best practice AH rehabilitation for any adult stroke sufferer was eligible for consideration. The 7-year search window encompassed the usual five-yearly update period for CPGs [16]. For inclusivity, guidance documents did not need to be called CPGs to be included. This is because different nomenclature has been reported internationally to describe similar types of guidance document [19]. Thus an inclusive, overarching term ‘guidance documents’ was used.

### Essential preliminary steps

To ensure the common understanding of purpose and an efficient focus for discussions, agreement was reached at the first meeting on the underpinning project premises (Additional file 1) and on the assumptions underpinning the decision to employ existing guidance documents as the evidence base (Additional file 1). Moreover, based on the project team’s familiarity with international stroke rehabilitation CPGs, it was assumed that no one guidance document would be identified that would answer all project questions.

### Scope, target group and purpose

The scope was the best-practice guidance for AH rehabilitation for acute or chronic stroke, in any public healthcare setting in SA. Over 90% South African stroke patients receive treatment in the South African public system [1, 2]. The target group was AH policy-makers, clinicians, managers, educators and researchers. Not in scope were the areas of stroke care in which AH do not play a role in SA (specific pre-hospital emergency care, specific hospital-based medical care to manage and stabilise acute stroke, pharmaceutical management (except where it may be relevant to AH rehabilitation)). Whilst AH care specific to the South African private sector was not directly in scope, guidance documents discussing private sector care may be included, if relevant.

The project purposes were to

1. Produce a methodology for efficiently finding and combining existing evidence sources into new, comprehensive, evidence-based, implementation-ready, locally-relevant guidance for resource-constrained healthcare settings, and
2. Test the methods by producing best-practice guidance for AH rehabilitation for South African stroke survivors from current international guidance documents.

The research built on the implementation framework proposed in the South African Guidelines Excellence Project (Project SAGE 2013–2017) [20, 21]. Project SAGE framework produced a three-tier model of CPG activity particularly relevant to resource-constrained environments. Tier 1 was a summary of the current best-available evidence; tier 2 reflected local stakeholder input on implementation of tier 1 evidence and tier 3 comprised documentation, collated from existing resources, or developed specifically for local contexts, to underpin efficient local implementation of tier 1 evidence [20].

### Identifying relevant guidance documents

A comprehensive search of internet repositories was undertaken to identify guidance documents for AH stroke rehabilitation published since January 2010, by any organisation, in any country. The search strategy is provided in Additional file 1.

### Guidance document quality

Irrespective of their nomenclature (CPG, guidelines, protocol etc) [19], methodological quality of the included guidance documents was assessed using the AGREE II instrument [22, 23]. The AGREE II domains are scope and purpose, stakeholder involvement, rigour of development, clarity of presentation, applicability, editorial independence. Because of its sensitivity, AGREE II would discriminate between the construction quality of different types of guidance documents [23]. For instance, it would detect differences in the rigour of development domain between an opinion-based protocol and a CPG based on systematic evidence searching [23]. The project team also assumed that the use of the AGREE II instrument overrode the need to interrogate included guidance documents for search strategies, literature inclusion, evidence synthesis methods, evidence tables or included studies.

Independent scorer dyads, experienced in using AGREE II, scored randomly-allocated guidance documents, and the scoring rubric was calculated independently using a MSExcel macro [22, 23]. Whilst AGREE II instrument metrics are not usually reported as total scores, these were calculated as an overall quality indicator. Arbitrary total overall quality score classifications were determined as

- high quality (HQ) being 80% + of the total possible AGREE II score,
- moderate quality (MQ) being 60–79% of the total possible AGREE II score and
- poor quality (PQ) being < 60% of the total AGREE II score.

### Project questions

The project team identified, discussed and agreed on key questions which, if answered by current best-evidence, could improve the quality of South African AH rehabilitation. A five-step process was taken.

**Fig. 1:**
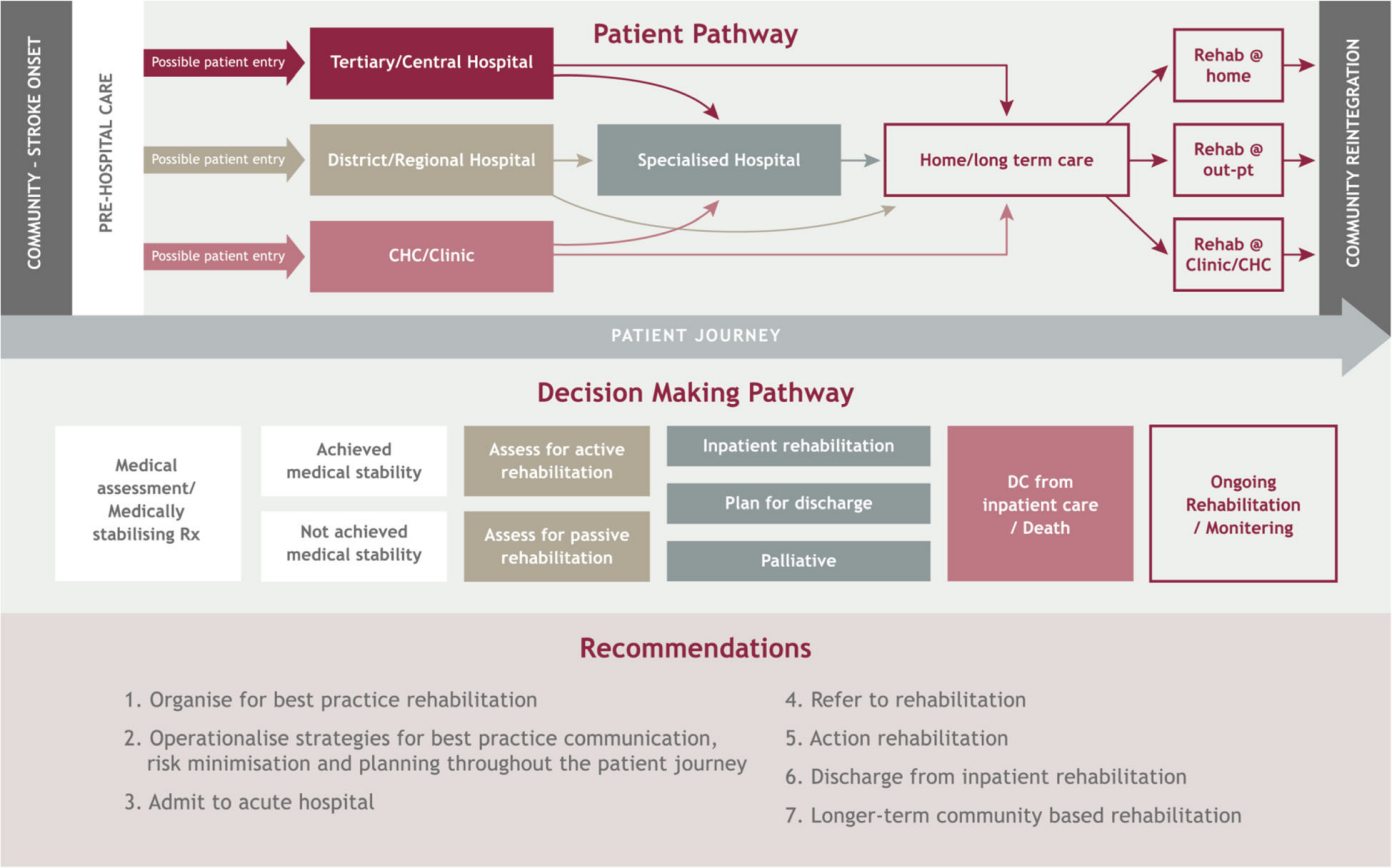
Patient journey through the South African public healthcare system, pathway of care decisions and relevance of recommendations to the decision-making pathway of care

1. ‘Usual’ patient care pathway(s) were established during the first project team meeting [24]. These outlined how, where and when stroke patients usually accessed care in the South African public sector [1, 2, 13]. South African stroke patients could enter and exit South African public sector healthcare at different points, as outlined in the top layer of Fig. 1. This pathway assisted in framing the project questions.
2. The project team then discussed AH rehabilitation activities related to the patient care pathways and raised issues for which there was variable practice and/or uncertainty about what to do. This process highlighted the complexities of delivering best-practice SA stroke rehabilitation, particularly the need for guidance on the workforce, training, organisation of services and communication. These issues are reported in the second layer of Fig. 1.
3. The team constructed 47 questions for which evidence-based answers were required. Questions were classified relevant to second tier of the patient pathway in Fig. 1. By this process, seven broad patient care activity clusters were identified, within which answers (recommendations) could be provided (see the third tier of Fig. 1). These clusters comprised organise for best practice rehabilitation, operationalise strategies for best practice communication throughout the patient journey, admit to acute hospital, refer to inpatient rehabilitation, action inpatient rehabilitation, discharge from inpatient rehabilitation and longer term community-based rehabilitation. This approach highlighted that some activities were relevant across the entire pathway (e.g. organisational and risk-minimisation activities), whilst activities such as referral to rehabilitation, actioning rehabilitation and discharge planning were specific to sections of the patient journey.
4. The project questions were condensed to 38 by team discussion (Table 1). These were organised by intent and relevance to the activities described in the second and third tier of the patient pathway (see Table 2). Eighteen questions related to communication, seven to service delivery, 15 to organisational issues, nine to clinical questions and five to training. Of note was the small number of questions related to clinical care. This validated the adoption of the WHO implementation focus, as it was evident that there was greater local need to know about ‘who’, ‘how’, ‘when’, ‘where’, ‘why’, ‘how much’, rather than about ‘what to do’ [18].
5. The project team anticipated that no one guidance document might answer all project questions. This was because of the specific nature of the South African stroke rehabilitation project but also because of the different purposes for which current international AH stroke rehabilitation guidelines might be written. Thus, if a guidance document identified in the search did not answer a project question, it was assumed that this question was not relevant to its scope or purpose.

**Table 1:** Project questions

1. Which factors might delay admission to medical facility after suffering a stroke at home? ^c^	
2. What is the optimal time for referral to rehab since admission to hospital? ^4,3,2,1^	
3. What is the optimal time for commencement of rehab since suffering a stroke? ^4,3,2,1^	
4. What are the factors indicating when it’s safe for rehab to commence? ^4,3,2,1^	
• EB assessment planning ^4,3,2,1^	
• Which factors should be assessed?	
• Which outcome tools should be used?	
5. Best practice recording method for assessment, treatment and goal setting when treating a stroke patient? ^4,3,2,1,h,p^	
6. What is critical to record when assessing and treating a stroke patient? ^4,3,2,1, h, s^	
7. What is the best, locally relevant communication platform for improving communication between levels of care; medical personnel; therapists; therapist/patient; therapists/family; therapist/employer? ^4,3,2,1, h, s^	
8. What should be communicated with medical personnel, other rehab therapists, patient and carer/family? ^4,3,2,1,h,p^	
9. What are the EB guideline on setting rehab goals and how to record these goals? ^4,3,2,1, h, s^	
10. EB discharge planning: ^4,3,2,1, h^	
- When should it start for a stroke patient?	
- Who should be involved?	
- What should it include?	
11. Which rehab professional should first see the patient? ^4,3,2,1, h^	
• What is the EB most critical first step?	
• What are the EB criteria for referral between therapists?	
• What is the best practice communication between therapists (devises, discharge planning and care continuation)?	
12. According to the evidence, which therapist should communicate with the family? ^4,3,2,1, h, s^	
13. What is the EB role of the physiotherapist, occupational therapist and speech therapist when assessing and treating a stroke patient? ^4,3,2,1^	
14. How does the model of care differ between the different points of entry (primary; secondary; tertiary; quaternary level)? ^4,3,2,1^	
15. What are the EB rehab interventions at each level of care? ^4,3,2,1, h, s^	
16. What are the best outcome measures for SA context for all levels of care as well as urban, suburban and urban settings? ^4,3,2,1, h, s^	
17. When should family education commence? ^4,3,2,1, h, s^	
• Which communication channel is most appropriate?	
• How is family incorporated into discharge planning?	
• Who should be communicating?	
• What should be included in the communication and in which format?	
18. What is the EB criteria for referral to other professions such as social workers/psychologists? ^4,3,2,1, h, s^	
19. Which rehab professional should take responsibility for planning and monitoring continuation of care? ^4,3,2,1, h, s^	
20. What are the EB rehab criteria for discharge from rehab as an in-patient and out-patient? ^4,3,2,1, h, s^	
21. What is the EB information for the best next level of care? ^4,3,2,1, h, s^	
22. What are the EB interventions for longer term care ^h, s^	
– rehab facility	
– Community Health Center (CHC)	
– long term home care	
– home or community	
23. What are the EB ways of communicating with patient/family/other professionals? ^4,3,2,1, h, s^	
24. What are the EB rehab outcome measures for longer term care? ^h, s^	
25. What is the EB education linked to complications of stroke (aspiration pneumonia/ secondary strokes etc.) ^4,3,2,1, h, s^	
26. How should Traditional healers be incorporated into the medical system? ^c, h^	
27. What training should traditional healers received to appropriately refer a stroke patient? ^c,h^	
28. What are EB criteria for ending rehab? ^h, s^	
• Ongoing monitoring?	
29. What is the evidence for the swallow test? When should it be done and by whom? ^4,3,2,1^	
30. What are the EB criteria for assistive technology? ^4,3,2,1, h, s^	
– Walking Aids	
– Slings	
– AFO’s	
– Wheelchairs	
– Splints	
– OT tools???	
31. What is the EB approach to re-integrating stroke patient into the community, society, leisure and work (participation)? ^h, s^	
32. How should rehab therapists liaise with other sectors (transport/labour/social) for facilitated participation? ^h, s^	
33. How should the community/general public be educated to facilitate societal participation of a person who has suffered a stroke? ^h, s^	
34. Therapists are not trained for inter-sectorial integration when it comes to general care/rights of a person who has suffered a stroke. What is the best practice to address this issue? ^h, s^	
35. “Work hardening”; aerobic capacity, effort and tolerance: ^4,3,2,1, h, s^	
- When should treatment or focus on these factors start?	
- What is the evidence based strategy to address this?	
36. Self-efficacy – compliance to medication and self-care:^4,3,2,1, h, s^	
- When should this start?	
- Which therapist should be responsible for educating patient?	
37. Best practice to work with mental health professionals or issues???? ^4,3,2,1, h, s^	
38. Best practice to equip/educate rehab therapist to deal with bereavement and depression after stroke? ^4,3,2,1, h,^	

Key: “c” refers to “Community”; “h” to “Home/long term care”; “s” to Society; “1” to “Primary”; “2” to “Secondary (District/Regional)”; “3” to “Tertiary”; and “4” to “Quaternary”

**Table 2:** Clusters of questions per intent for implementation purposes

	1	2	3	4	5	6	7	8	9	10	11	12	13	14	15	16	17	18	19	20	21	22	23	24	25	26	27	28	29	30	31	32	33	34	35	36	37	38
Communication		x			x	x	x	x	x	x	x						x	x		x	x	x	x				x					x	x			x		
Service delivery			x														x	x		x	x	x									x							
Organisational										x	x	x	x					x	x	x	x	x				x	x			x	x	x				x		
Clinical				x											x	x									x			x	x	x	x				x			
Training requirements									x			x																						x			x	x

### Excluding guidance documents

The scope, purpose and questions addressed in the guidance documents identified in the search were mapped to the South African project questions. To be included, a guidance document should answer at least one South African project question, provided in the form of a recommendation (this is defined in a later section). Poor methodological quality was not a reason for exclusion, as quality was taken account in the calculation of the overall strength of the body of evidence (OSoBE) (see later section).

### Strength of the body of evidence (SoBE)

A summary of the relevant evidence usually underpins recommendations in good quality guidance documents evidence, reported as SoBE [25, 26]. Berkman et al. [27] define SoBE as a method ‘to help clinicians, policymakers, and patients make well-considered decisions about health care. The goal of strength of evidence assessments is to provide clearly explained, well-reasoned judgments about reviewers’ confidence in their systematic review conclusions so that decision-makers can use them effectively’ (p 1314). However, there is no standard approach to formulating or reporting SBE, which presents a challenge when comparing recommendations from different guidance documents.

### Defining recommendations

There was no standard way of defining a ‘recommendation’. Expanding Alper’s [28] and Schiffmans work [29], recommendations in this project were defined as

- wording in guidance documents that was clearly labelled ‘recommendation’ (appearing in designated recommendation boxes, specific fonts or tables) and accompanied by a SoBE grading, or
- wording that appeared in the text, not necessarily labelled ‘recommendation’ but which had the intent of being a recommendation, in terms of intention words such as ‘should’, ‘could’, ‘might consider’, accompanied with an SoBE grading.

Not considered as recommendations were words which appeared in the body of guidance documents which were not labelled ‘recommendation’, did not have the intent of a recommendation or had no SoBE grading.

### Extracting data for project questions

Separate purpose-built data extraction sheets were developed for each project question. These recorded the details of the guidance documents which answered it (year of construction, methodological quality, country of origin), recommendations relevant to the question, and the SoBE gradings for recommendations (in whichever way they were reported). Moreover, any guidance document which provided ‘how to do it’ information was identified as potentially providing useful tier 3 documents. These could include, but were not limited to, protocols, patient management or service decision-making tools, organisational flowcharts, stroke team construction, workforce issues, assessment criteria, specific assessment tools, outcome measures, minimal clinically significant changes from interventions, discharge planning checklists and patient information material.

### Standardising SoBE

A common, readily interpreted set of ‘faces’, was developed for this project to standardise the different ways in which SoBE was presented in included guidance documents. A smiley face was used for positive evidence, a neutral face for insufficient or conflicting evidence and a frowning face for negative evidence. Details are provided in the ‘Results’ section.

### Compiling the evidence

A summary table was developed from the individual data extraction files, reporting the guidance documents which provided answers to each project question (volume of evidence), year of production of guidance documents (currency of evidence), their methodological quality and the consistency of recommendations (‘do all recommendations point in the same direction’?). Project questions were then classified as

- those which were not answered by any guidance document (absence of evidence),
- those answered by only one–two guidance documents (scant evidence) and
- those with inconsistent SoBE (inconsistent evidence).

### Writing composite recommendations

There is currently no methodology about how to develop a composite recommendation which summarises the intent of recommendations extracted from two or more guidance documents. The project team developed a new approach using inductive thematic content analyses within a social phenomenology paradigm to do this [30, 31]. Schutz [31] proposed social phenomenology as a descriptive, interpretive theory of social action using ‘*subjective experience within the* “*taken-for-granted, commonsense*” *world of the daily life of individuals*’ and takes the view ‘*that people living in the world of daily life are able to ascribe meaning to a situation and then make judgments. It is the subjective meaning of experience that was the topic for interpretation in this study*’ (cited by Fereday & Muir-Cochrane [32] (p.81)). By taking this approach, project team members’ broad understanding of aspects of SA AH stroke rehabilitation could be aligned with the intent of international stroke rehabilitation recommendations. These discussions also provided evidence credibility trails [27–31, 33].

For each project question, researcher dyads used an inductive analysis approach and discussed and compared wording, content, intent and meaning of recommendations extracted verbatim from guidance documents. Draft composite recommendations for each question were proposed to the other members of the project team, which discussed and ratified them for relevance to South African contexts.

### Determining the OSoBE for composite recommendations

There is an emerging body of methodological guidance about how to develop OSoBE gradings for composite answers distilled from multiple CPG recommendations. To assign OSoBE gradings, the project team drew from

- the determination methods of Gonzalez-Suarez et al. [24] (consistency of thought, volume of evidence, strength of evidence),
- the evidence grade elements of the NHMRC strength of the body of evidence matrix [25] (the evidence base, consistency and impact of recommendations),
- the decision-making flowchart proposed by Alper et al. [28] to reconcile recommendations from a small number of CPGS which address the same question, based on consistency of findings, and reported SoBE gradings [34] and
- the GRADE approach, used in de novo CPG construction of moving from evidence to recommendations [35, 36].

**Fig. 2:**
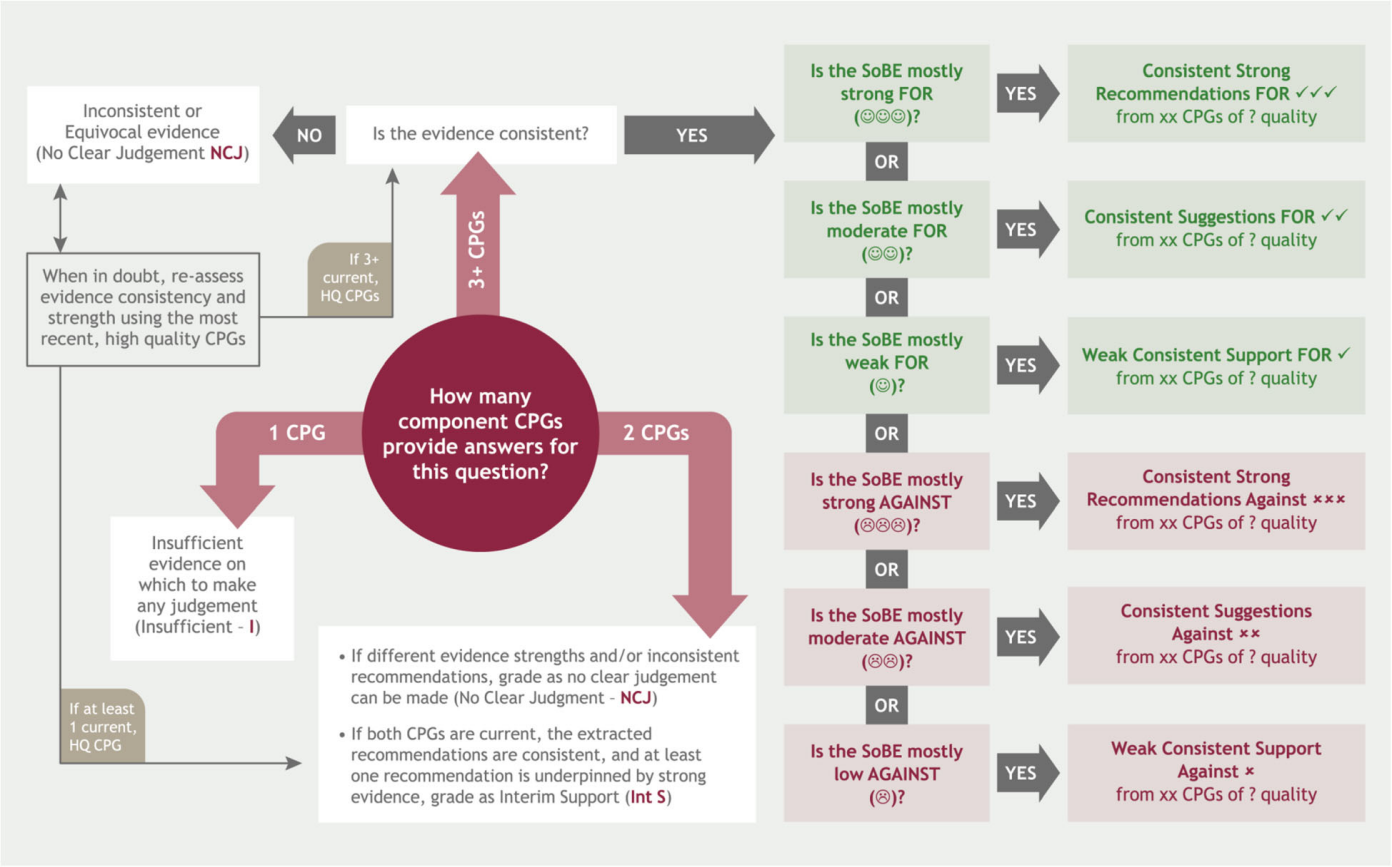
Decision-making process for determining the OSoBE for each composite recommendation

A decision-making framework to determine OSoBE was developed and tested (Fig. 2). This included the SoBE grades from the component guidance documents for each question and the number, consistency, quality and currency of these documents (See steps outlined in Additional file 1).

**Fig. 3:**
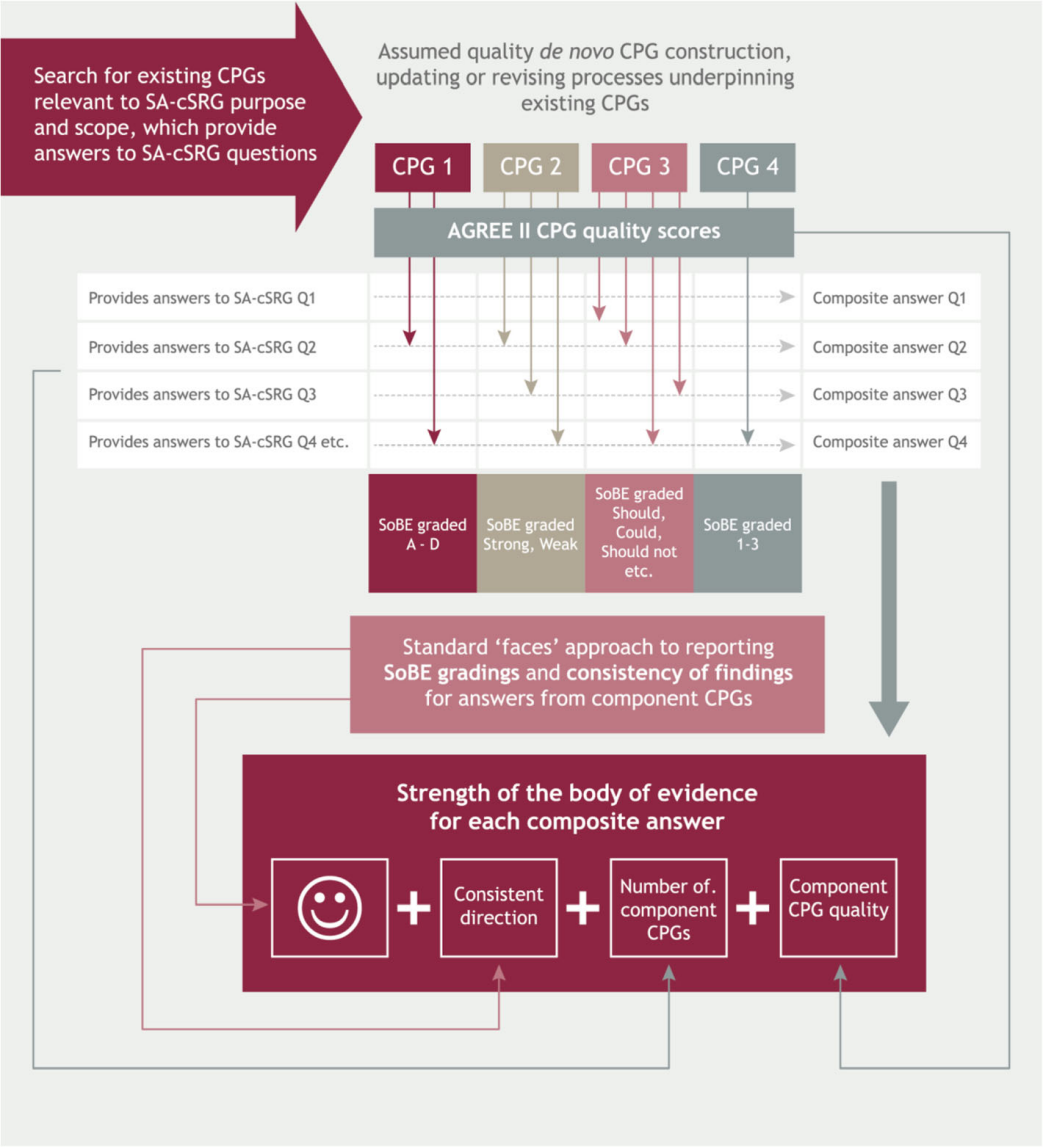
Conceptual framework for the processes undertaken in this project

A conceptual framework outlining the project methods is summarised in Fig. 3.

### Endorsement of process and recommendations

Regular feedback and workshops supported the development and validation of the evidence-synthesis processes and endorsement of composite recommendations and OSoBE.

## Results

### Included guidance documents

The search identified 16 guidance documents [37–52] (See Table 3). Each document had been named as a ‘Clinical (Practice) Guideline’, although purpose, scope, questions, methodology, presentation, layout and content differed. All 16 guidance documents were included because they answered at least one project question (See Table 1). The broad interest in AH stroke rehabilitation in upper-middle and high-economic countries was reflected by the volume of documents and countries producing them. Four documents each came from Australia, USA and UK, and one each came from Canada, Malaysia, South Africa and New Zealand. None came from low or lower-middle-income countries.

**Table 3:** Included guidelines, evidence sources and total AGREE score

Clinical practice guideline	Acronym	Year	Source of evidence	AGREE score
American Heart Association/American Stroke Association (rehabilitation guideline)	AHA/ASA	2015	Relevant articles on adults using computerised searches of the medical literature through 2014	64.9% (mod)
American Occupational Therapy Association (sourced through Guidelines Clearing House AHRQ)	AOTA	2013	Databases and sites searched included Medline, PsycINFO, CINAHL, AgeLine, and OTseeker, consolidated information sources(Cochrane Database of Systematic Reviews, Campbell Collaboration); reference lists from articles included in the systematic reviews were examined, and selected journals were hand searched	72.5% (mod)
American National Guidelines Clearing House summary (sourced through AHRQ)	AHRQ	2013	Commissioned by and extracted from NICE rehabilitation guidelines (2013) (based on NICE methods). A panel of independent multidisciplinary experts debated the findings and wrote recommendations	73.2% (mod)
Australian Council on Safety and Quality in Health Care	ACSQHC	2015	Australian clinical practice guidelines, standards and policies were identified from • the clinical practice guideline portal of the National Health and Medical Research Council (NHMRC) • websites of professional colleges and organisations • websites of state and territory health departments and agencies • internet search using various search engines. International clinical practice guidelines • guideline clearing houses such as the Agency for Healthcare Research and Quality (AHRQ) and Guidelines International Network (GIN) • websites of guideline developers, such as the UK’s National Institute for Health and Care Excellence (NICE) and Scottish Intercollegiate Guideline Network (SIGN). Other high-level evidence was identified by searching • the Cochrane Collaboration for systematic reviews and meta-analyses • medical literature databases (Medline, Embase) for systematic reviews and meta-analyses.	27.5% (poor)
Australian Stroke Foundation	ASF	2017	Update evidence search using primary and secondary literature from library databases, Cochrane Collaboration, high quality international guidelines NB search strategy similar to all previous versions of ASF guidelines	63.0% (poor)
Canadian stroke guidelines	CSG	2015	Methods based on the Practice Guideline Evaluation and Adaptation Cycle (PGEAC) [54]. Systematic literature search w for each topic area by independent contract methodologists. Updated literature searches built on previous reviews from 2012 to 2015, which overlapped the previous search time frame by 6 months to ensure high catchment of key articles within that time frame. The writing group was provided with comprehensive evidence tables that include summaries of all high-quality evidence identified through the literature searches.	85.1% (high)
Dept. of Defence, Veterans Association Management of Stroke Guidelines	VA/DoD	2010	Recommendations for the management of stroke rehabilitation were derived through a rigorous methodological approach: • Determining appropriate criteria such as effectiveness, efficacy, population benefit, or patient satisfaction • Reviewing literature to determine the strength of the evidence in relation to these criteria • Formulating the recommendations and grading the level of evidence supporting the recommendation Findings were reviewed by an expert working party and recommendations produced relevant to veterans’ needs.	74.6% (mod)
Malaysian stroke guideline		2016	A panel of committee members was appointed comprising of neurologists, a cardiologist and a radiologist from the Ministry of Health, universities and the private sectors. Authors from the first CPG were invited to contribute on new updates before being discussed by panel members. The discussion started from early 2010 before being finalised and sent for the appointed reviewers. The group members met several times throughout the development of the guideline. All retrieved literature were appraised by individual members and subsequently presented for discussion during group meetings. All statements and recommendations formulated were agreed collectively by members of the Expert Panel. Where the evidence was insufficient the recommendations were derived by consensus of the Panel. The draft was then sent to local external reviewers for comments. The level of recommendation and the grading of evidence used in this guideline was adapted from the U.S./Canadian Preventive Services Task Force and the Guidelines for Clinical Practice Guideline, Ministry Of Health Malaysia 2003. The principles and layout follows the methodology stated in the Guidelines for Clinical Practice Guidelines booklet published by the Medical Development division of the Ministry of Health Malaysia. A standard methodology based on a systematic review of current evidence was used to look at the literature. These guidelines have been presented to the Chairman of the Health Technology Assessment and Clinical Practice Guidelines Council of the Ministry of Health Malaysia for review and approval.	81.9% (high)
New Zealand Guidelines Group	NZGG	2010	Builds on the ASF search 2009. Systematic identification of relevant studies was conducted between May and August 2009, EMBASE, Medline and Cochrane databases were used. CINAHL and Psychinfo databases were searched where relevant. The PEDro database was used to check PT studies. A second updated search of the literature up to 19 February 2010 using Medline and EMBASE databases was conducted. Updated Cochrane reviews were also searched and included. Economic studies were included where available	73.6% (mod)
National Institute for Health and Clinical Excellence	NICE	2013	De novo literature reviews were undertaken including evidence from economic studies, consensus was sought on the evidence findings for each question	75.4% (high)
NSW Agency for Clinical Innovation	NSW ACI	2016	2015 National Acute Stroke Services, Framework by the National Stroke Foundation; 2015 Acute Stroke Clinical Care Standard and Indicator Specification by ACSQHC; 2010 Clinical Guidelines for Stroke Management NSF; 2015 Focused Update of the 2013 Guidelines for the Early Management of Patients with Acute Ischemic Stroke Regarding Endovascular Treatment by the American Heart Association/American Stroke Association; NSW ACI Stroke Reperfusion Program Evaluation Report; 2015; Bureau of Health Information, The Insight Series, 30-day mortality following hospitalisation, five clinical conditions. July 2009–2012 (7); Middleton S et al. ‘Implementation of evidence-based treatment protocols to manage fever, hyperglycaemia, and swallowing dysfunction in acute stroke (QASC): a cluster randomised controlled trial’ Lancet 378(9804): 1699–706.(20)	65.6% (mod)
Royal College of Physicians	RCP	2012	Systematic searching of computerised databases Medline, AMED, CINAHL, Psychinfo and Embase. The Cochrane Collaboration database, SIGN and NICE; Health Technology Appraisal (HTA) reports; members of the working party brought their own expertise and information from their organisations and professional bodies. For topics newly added since 2008 searches included the time period from 1966 onwards; for the remainder of the topics searches were performed from 2007 until February 2012.	90.2% (high)
South Australian Dept. of Health Stroke Network	SA Dept. of Health SN	2017	Expert input	79.0% (mod)
Scottish Intercollegiate Guidelines Network (dysphasia, rehabilitation)	SIGN	2010, 2010	De novo and updating searches as per all SIGN activities (comprehensive systematic reviewing, critical appraisal, independent data extraction)	81.5% (mod)
South African Stroke Society	SASS	2010	Consensus based on AHA/ASA guidelines	51.8% (poor)

### Questions and composite recommendations

No guidance document answered all project questions, thus, we found no comprehensive existing source of evidence-based guidance for AH rehabilitation that could be immediately applied to guide South African AH stroke rehabilitation in any SA healthcare setting. Overall, the guidance documents addressed all but five project questions.

### Methodological quality

Methodological quality varied (See Table 3 for overall AGREE II scores). Four guidance documents were of high quality [42, 44, 46, 48], eight were moderate quality [37–39, 43, 45, 47, 49–51] and three were poor quality [40, 41, 52]. The age of the guidance was not correlated with methodological quality scores (*p* > 0.05). The AGREE II domain scores are reported in Additional file 1. Considering the domains which provided most relevant information on transferability of guidance documents to the South African AH stroke rehabilitation questions, for scope and purpose, all but one guidance document scored ≥80% (New Zealand [45] which scored 77.8%). For rigour of development, only three guidance documents did not score highly. These comprised two with poor (≤ 30%) scores (ACSQH [40], South Africa [52]), whilst the third (AHA/ASA [41] scored moderately (72.9%).

### Standardising SoBE gradings

As anticipated, the included guidance documents reported different ways of determining and reporting SoBE gradings. Table 4 outlines these and the standardised SoBE approach developed for this project.

**Table 4:** Ways in which the SoBE gradings were reported in component CPGs and the SA-cSRG standard ‘faces’ system

NICE/ AHRQ^[1]^ (consensus wording from evidence strength)	VA/ DoD	RCP (consensus on evidence strength), Sth Aust; NSW ACI	AHA/ASA, Canada^[2]^, Sth Africa^[3]^	NZGG^[4]^, Malaysia, SIGN	ASF	SA-cSRG standard approach
Evidence synthesis and consensus words	Hierarchy and quality	Evidence synthesis and consensus words	Hierarchy and quality	Hierarchy and quality	Evidence strength words	
High confidence (positive)	A	Strong = should	A	A	Strong	☺☺☺
Moderate confidence	B	Moderate = could	B	B	Strong	☺☺
Low confidence	C	weak = apply with caution	C	C	Weak	☺
	D			D	Weak	☺
	Insufficient					
			Opinion	Practice point	Practice point	☺ or ☹
Moderate confidence (negative)		Moderate not				☹☹
High confidence (negative)		Should not		A	Strong (against)	☹☹☹

^[1]^After results were pooled, the overall quality of evidence for each outcome was scored using GRADE (NICE p46):

• A quality rating was assigned, based on the study design. RCTs start HIGH and observational studies as LOW, uncontrolled case series as LOW or VERY LOW

• The rating was then downgraded for the specified criteria: study limitations, inconsistency, indirectness, imprecision and reporting bias. These criteria are detailed below. Observational studies were upgraded if there was a large magnitude of effect, dose-response gradient and if all plausible confounding would reduce a demonstrated effect or suggest a spurious effect when results showed no effect. Each quality element considered to have ‘serious’ or ‘very serious’ risk of bias was rated down 1 or 2 points respectively

• The downgraded/upgraded marks were then summed, and the overall quality rating was revised. For example, all RCTs started as HIGH and the overall quality became MODERATE, LOW or VERY LOW if 1, 2 or 3 points were deducted respectively

• The reasons or criteria used for downgrading were specified in the footnotes

^[2]^Canadian stroke best practice recommendations overview and methodology documentation available on the Canadian stroke best practices website at www.strokebestpractices.ca

^[3]^Based on AHA recommendations and classifications

^[4]^Based on earlier versions of Aust Stroke Foundation CPGs (ASF 2008–2010)

NB: ASCHC did not provide any evidence strength; it reported only those guidelines that supported its summary recommendations

**Fig. 4:**
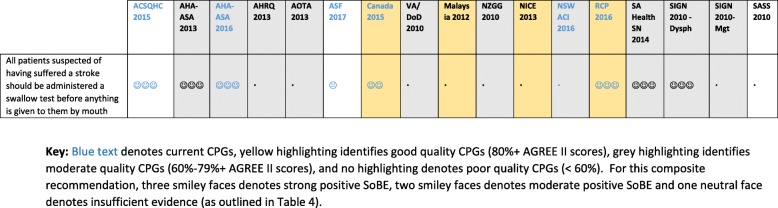
An example of the standardised SoBE assigned to recommendations extracted from guidance documents which answered questions 2, 3 and 4 (See Table 1 for questions; see Additional file 1 for guidance document recommendations and decision-making steps)

An example of a SoBE summary for three project questions (2, 3 and 4) is provided in Fig. 4. This figure includes the initial SoBE grading for recommendations relevant to these questions, the standardised SoBE, and the consistency of the evidence (number and type of ‘faces’). Additional file 1 outlines the steps taken to answer the three project questions (2, 3 and 4).

### Composite recommendations

Most of the composite recommendations were underpinned by moderate to good OSoBE, which provided believable tier 1 evidence to support contextualised implementation of recommendations into SA healthcare settings. Additional file 1 reports the composite recommendations clustered into the patient care decision-making categories (third tier Fig. 1) and the relevant OSoBE.

## Discussion

This is the first research of which we are aware that has developed and tested processes to transparently summarise recommendations from multiple guidance documents, to answer questions relevant to a local context problem. Our project builds on the research reported by Alper [28] and Shiffman [29] in terms of combining recommendations from multiple evidence sources, and using strength of wording in recommendations to underpin implementation decisions.

The new methods address the lack of guidance in the literature about how to

- standardise differently reported SoBE gradings,
- synthesis intent and wording from multiple recommendations that reported the evidence for one question in different ways and
- determine an OSoBE grading for a composite recommendation which summarised recommendations from multiple guidance documents, for one question.

The test vehicle for this work was AH stroke rehabilitation delivered in a country (South Africa) that cannot afford the time or finances to develop its own de novo CPG. Moreover, because of the burden that stroke imposes on the SA economy, on communities, families and individuals, AH stroke rehabilitation in SA needs best evidence recommendations to ensure that scant resources are used wisely and equitably [1, 2, 13, 15]. Thus, it was an ideal vehicle to test the new methods.

These new methods add to discussions on methods of CPG repurposing and transferability from one context to another. The methods should assist other South African CPG teams and teams in other resource-constrained organisations or countries, to efficiently contextualise currently available guidance documents for other health conditions. Our work provides a simple, new step-by-step approach that focuses efforts on evidence implementation, rather than on development of de novo CPGs [26], when there was no urgent need to do so. We believe that the key to the project was the development of a clear and comprehensive patient pathway (pioneered in the Philippines [24]). This pathway provided a visual overview of the public healthcare options for stroke rehabilitation in SA and provided an important prompt for discussions regarding what actually happened prior to, during and after AH stroke rehabilitation for average and atypical patients with stroke. This assisted the project team to formulate questions. The best practice recommendations now linked to this pathway have the potential to impact on policy, since the National Health Insurance (NHI) of South Africa white paper [53] strongly supports the use of local CPGs to guide the delivery of evidence-informed and cost-effective health services in SA.

Project team members with little background in methodology found the step-by-step process simple to understand, and they required little further explanation than was provided in the figures reported in this paper. The simplicity of the process meant that future work should be able to be undertaken with minimal training by non-academics (clinicians, managers, policy-makers) or even students. Those managers and clinicians on the project team who were less focused on methodology, and more interested in recommendation relevance and implementation in local settings, validated the usefulness of the wording and intent of the composite recommendations, as well as the way the OSoBE had been developed. They indicated that the recommendations aligned with their understanding of current best practice undertaken elsewhere in the world. The policy-makers expressed confidence that the composite SA–relevant recommendations positioned them to progress evidence-informed discussions on future funding and policy priorities. Consumers considered that the process encouraged engagement of consumers on future guidance teams. The recommendations would also improve SA patient understanding of rehabilitation, as well as shared decision-making.

Integrating existing recommendations was far more efficient than starting ‘from scratch’ with a de novo CPG oriented to South African contexts [25, 26]. This project was conducted with minimum of external funding or reliance on individual ‘out-of-hours’ commitment. Project team members’ employers largely agreed to commitment to this project within work hours. The team members came from different parts of South Africa, thus, it took time and resources to organise face-to-face meetings. Much contact occurred electronically, when versions of recommendations were being discussed. Development of the process and constructing the new evidence base from 16 guidance documents took approximately 3 months intensive work by the research team, compared to 12–18 months to produce a de novo CPG. There were four team meetings (1 day each), and the remainder of the project time was spent in gaining agreement and endorsement of recommendations using electronic means. It is anticipated that future applications of the new methods to produce composite guidance for other health conditions in other settings would be quicker, particularly if there are fewer relevant guidance documents to summarise.

The need for this project was highlighted as there was no one international CPG which answered all the project questions. Moreover, the project questions contained few clinical ones (‘what to do’) and mostly focused on implementation of evidence locally (‘who’, ‘how’, ‘when’, ‘where’, ‘why’ and ‘how much’). This validated the theoretical model proposed by Project SAGE [20], of using three CPG tiers when constructing local guidance. The use of existing ‘what to do’ recommendations extracted from existing guidance documents provided the current best evidence base (tier 1) for AH stroke rehabilitation potentially relevant to South African public healthcare sectors. What is more, the extracted information supported effective and efficient tier 2 discussions on local service delivery implementation issues. The majority of included guidance documents were well constructed (moderate to good AGREE II scores). Their scope and purpose correlated with at least some of the project questions, making these guidance documents relevant evidence sources. Useful tier 3 documents were also identified from the included guidance documents, obviating the need to reproduce these locally. Whilst the SoBE gradings varied between guidance documents for the recommendations pertinent to the project questions, the evidence was mostly consistent in terms of consistency of direction, intent and wording.

The importance of including the multiple components in the OSBoE decision-making framework was highlighted by the variability in the number and currency of included guidance documents providing answers to project questions. The differing SoBE gradings for extracted recommendations relevant to a project question mostly related to the underpinning research design(s) which formed the evidence base. This reflects a fundamental issue with determining appropriate SoBE gradings related to AH stroke rehabilitation. The notion of SoBE has its genesis in intervention studies, largely delivered using a medical model [25, 26, 35, 36, 54, 55]. High SoBE gradings generally come from prospective comparative studies with control arms and blinding (experimental trials, diagnostic studies etc.). High quality intervention studies are often difficult to design for AH stroke rehabilitation because lack of homogeneity in stroke aetiology, affected site and stroke density, access to subjects, individuals’ responses to brain insult, attitudes to therapy, service delivery differences, family and community support, potential for quality life after stroke etc. [12, 15, 55]. Moreover, service quality research questions are mostly answered by cross-sectional or retrospective designs which usually graded as having moderate to high risk of bias and, therefore, lower SoBE grades [35, 36, 55]. In SA, where stroke service delivery can differ markedly across public health service sectors, and in urban, regional and remote areas, there is no one clear patient care pathway for many stroke sufferers from acute care to long-term rehabilitation and community integration. This was why the discussions on the patient pathway were so valuable at project commencement (See Fig. 1).

The applicability of our novel method requires further testing by others, in other healthcare settings, for stroke and other health conditions. The proposed methods to combine recommendations and SoBE gradings need validation in different types of research evidence. However, critical to update of this method is the issue of transferability of recommendations from existing CPGs to new environments. This must be guided by tier 2 (local expert input). Evidence that has been developed on one population may not be immediately transferrable to other populations, even if patients come from similar socioeconomic environments [24, 28, 29]. Implementation-focused questions need to be asked such as ‘Can this recommendation be put into place for our patients, in our contexts’, and if the answer is no, then the reasons for this need to be explored in terms of local barriers and facilitators. Getting the right tier 2 representatives together and engaging them in discussion relevance of recommendations appears to be key to moving from evidence-based recommendations to implementation-ready recommendations.

The limitations were that a new process was developed and tested on one set of questions (allied health stroke rehabilitation). This condition provided a vehicle. The project team was learning as it went, and errors and mis-interpretations were sure to have been made, particularly in the formulation of composite recommendations from the included guidance documents. The process of combining evidence sources into one composite set of recommendations needs to be tested by other teams for other conditions.

## Conclusion

Our practical innovative methods add to the scarce body of evidence in repurposing and transferring CPGs from one etting to another. Given the urgent need to implement evidence in resource-constrained settings to reduce alarming disease burdens, the costs and time to develop de novo CPGs, and the untenable burden the lack of appropriate evidence places on many low- and lower-middle economy countries, our methods provide a feasible, efficient process to repurpose and transfer CPGs, rather than produce new ones. The SA AH stroke rehabilitation recommendations developed using this new method will ensure that appropriate treatments are offered at appropriate times, in appropriate ways, relevant to local contexts, to improve stroke survivors’ access to ongoing care and quality post-stroke lives.

## Additional file

Additional file 1: Underpinning premises for this study. (DOCX 55 kb)

## Abbreviations

CPGs: Clinical practice guidelines
OSoBE: Overall strength of the body of evidence
SA: South Africa
SA-cSRG: South African contextualised stroke rehabilitation guidelines
SoBE: Strength of the body of evidence
WHO: World Health Organization
