# Supplementary material for: Standardising evidence strength grading for recommendations from multiple clinical practice guidelines: a South African case study
Source: Implement Sci. 2018 Aug 29;13:117. doi: 10.1186/s13012-018-0803-0 (PMC6114483; doi:10.1186/s13012-018-0803-0)
Supplement: Supplementary file 1 — Underpinning premises for this study. (DOCX 55 kb) [file 13012_2018_803_MOESM1_ESM.docx]

**APPENDIX 1. Agreed project premises**

1. There was no need to write *de novo* CPGS for stroke rehabilitation for South Africa, as many CPGS were already available internationally which efficiently summarised the current evidence base.
2. It was essential that the SA-c SRG group’s efforts focused on implementation of best available evidence to rehabilitation providers dealing with South Africans with stroke
3. The SA-c SRG project needed to address local care decisions, specific to an agreed ‘average’ patient pathway related to stroke rehabilitation in South African healthcare settings
4. The evidence for Tier 1 (the research evidence) would come from
   1. recommendations extracted from freely- and publically-available CPGs published since 2010 for stroke rehabilitation, that addressed the SA-c SRG questions, and
   2. the component CPG strength of the body of evidence (SoBE) grading underpinning each extracted recommendation. SoBE is defined in different ways in the literature, but largely deals with the confidence that end-users place in the quality with which the research was conducted, to provide a believable answer. Higgin et al (2011) note that *‘The notion of study “quality” is not well defined but relates to the extent to which its design, conduct, analysis, and presentation were appropriate to answer its research question’*.
5. No attempt would be made to interrogate the studies underpinning the included CPG recommendations, or to search for new primary or secondary literature where evidence gaps were identified (i.e. where there was no recommendation from current CPGs for a SA-c SRG question).
6. The component CPG SoBE grading for each extracted recommendation would be initially reported in the manner presented in the included CPG. It was anticipated that the component CPG SoBE gradings would differ depending on CPG construction methods. However it was also recognized that the different methods of SoBE grading would need to be standardized, as this element was essential for determining the overall strength of the body of evidence for composite recommendations
7. To provide a standard way of interpreting component CPG SoBE gradings, a ‘faces’ model was developed. This approach also assisted in determining consistency of findings (if all ‘faces’ were positive for instance, it would indicate that despite different ways of reporting SoBE gradings, the evidence all pointed in the one direction)
8. Composite recommendations (summary answers) for each SA-c SRG question would be constructed, using the intent and best choice of words from the component CPG recommendations relevant to each SA-cSRG question.
9. An overall SoBE (**OSoBE**) grading underpinning each composite recommendation (overall answer) for each SA-c SRG question would be established based on the Alper et al (2016, 2017) model, using the metrics of the standardized ‘faces’ system; consistency of direction of the ‘faces’; the number of component CPGs answering each question; their methodological quality; and (where indicated), their currency.

**APPENDIX 2. Assumptions underlying the use of recommendations from existing CPGs**

1. There was a clearly defined process outlined in each included component CPG, by which the CPG questions had been established (re scope and purpose as outlined in AGREE II);
2. The scope and purpose of the component CPGs, and the information their provided, mapped in part to the scope, purpose and questions of the SA-cSRG;
3. Each question addressed in component CPGs had been appropriately framed to find the best available evidence through literature searching (PICO, PICOS, PECOT etc);
4. The search strategies in the component CPGs had been accurately mapped to their CPG questions (i.e. intervention questions were answered by intervention or prospective cohort studies; diagnostic questions were addressed by diagnostic studies etc);
5. The included literature in each component CPG was current, and was comprehensively and systematically identified by defensible search terms and search strategies;
6. The methodological quality of the included literature in each component CPG had been appropriately interrogated to determine SoBE;
7. The ways in which data had been extracted and synthesized from the included literature, for each question addressed in each component CPG, was defensible, transparent and available for scrutiny; and
8. The ways in which the underpinning evidence in each component CPG had been synthesized and interpreted as recommendations, were comprehensively-described and defensible.

**APPENDIX 3. Search strategy**

*Search terms:*

| P | stroke OR cerebral vascular accident OR ischaemic stroke OR CVA |
| --- | --- |
| I | Rehabilitation provided by any allied health (AH) provider of any discipline |
| C | none |
| O | Any rehabilitation outcome (i.e. function, quality of life) |
| S | clinical practice guideline (OR guidance OR clinical guideline OR management protocol OR protocol OR algorithm OR patient management tool); |

*Timeframe:* 2010-2017

*Data sources:* Systematic general searches were conducted through [*www.google.com*](http://www.google.com), and specific searches were also conducted through international CPG clearing houses and CPG developers’ websites, including, but not limited to, Guidelines International Network (<http://www.g-i-n.net/home>) National Guidelines Clearing House *(*[*https://www.guideline.gov/*](https://www.guideline.gov/)*);* Scottish Intercollegiate Guidelines Network (SIGN) (UK) [*www.sign.ac.uk/*](http://www.sign.ac.uk/); National Institute of Health and Care Excellence (NICE) (UK) [*https://www.nice.org.uk/*](https://www.nice.org.uk/); National Health and Medical Research Council (NHMRC) (Australia) [*www.NHMRC.gov.au/*](http://www.NHMRC.gov.au/); New Zealand Guidelines Group (NZGG) ([*www.nzgg.org.nz/*](http://www.nzgg.org.nz/)*).*

**APPENDIX 4. Decision-making framework for overall strength of the body of evidence*.***

A detailed decision-making framework was developed to determine the overall strength of the body of evidence for each composite recommendation

***Considering Positive Evidence (FOR)***

- ***Strong Consistent Evidence For (a care action):*** When the composite recommendation is underpinned by three or more component CPG recommendations that have **high SoBE grading,** and provide **positive consistent recommendations for** (a care action) [☺☺☺], the composite recommendation wording states: ‘There are Consistent and Strong Recommendations from *xx* CPGs (*aa* high quality (HQ), *bb* moderate quality (MQ), *cc* (Poor Quality (PQ))’ **For** (a particular care action). This composite recommendation strength of the body of evidence has three ‘ticks’✔✔✔.
  - The evidence body strength could still be called ‘strong’ if it included
    - <10% individual moderate [☺☺] SoBE gradings for (a care action) considering all included CPGs; OR
    - moderate [☺☺] SoBE gradings underpinning recommendations for (a care action) from component CPGs older than five years, which may not have had the benefit of including new more definitive literature.
      - The percentage of older CPGs in the evidence dataset which met this rule was limited to 50% or less. If the percentage of older CPGs with moderate [☺☺] SoBE gradings was higher than 50%, the composite strength of the body of evidence was downgraded to ✔✔
  - However, if the moderate evidence [☺☺] SoBE gradings were reported for recommendations extracted from one or more recent, high quality component CPGs, the Alper et al (2017) decision-making algorithm would be invoked, and the composite strength of the body of evidence would be downgraded to ✔✔. The reasons for downgrading the composite strength of the body of evidence would be explained.
- ***Moderate Consistent Evidence For (a care action):*** When the composite recommendation is underpinned by three or more component CPG recommendations that are supported by **moderate SoBE gradings,** and provide **positive consistent recommendations for** (a care action) [☺☺], the composite strength of the body of evidence wording states: ‘There are Consistent Suggestions from *xx* CPGs (*aa* high quality (HQ), *bb* moderate quality (MQ), *cc* (Poor Quality (PQ)) **For** (a particular care action)’ The composite recommendation strength of the body of evidence has two ‘ticks’ ✔✔
  - The evidence body strength could still be called ‘moderate’ if it included
    - <10% low SoBE gradings [☺]overall from the included CPGs; OR
    - Low SoBE gradings [☺] for recommendations from included component CPGs older than five years, which may not have had the advantage of including new, more definitive literature
      - The percentage of older CPGs in the evidence dataset which met this rule was limited to 50% or less. If the percentage of older CPGs with low [☺] SoBE gradings was higher than 50%, the composite strength of the body of evidence would be downgraded to ✔
  - However, if the low SoBE grading [☺] came from one or more recent, high quality component CPGs (published within the last five years), the Alper et al (2017) decision-making algorithm would be invoked and the composite strength of the body of evidence would be downgraded to weak ✔. The reasons for downgrading the composite strength of the body of evidence would be explained.
- ***Weak Consistent Evidence For (a care action):*** When the composite recommendation is underpinned by component CPG recommendations that are generally underpinned by **low SoBE gradings,** but provide **positive consistent recommendations for** (a care action) (☺), the composite strength of the body of evidence wording states: ‘There is Weak Support from *xx* CPGs (*aa* high quality (HQ), *bb* moderate quality (MQ), *cc* (Poor Quality (PQ)) **For** (a particular care action)’. The composite recommendation has one ‘tick’ ✔
  - The evidence body strength could still be called ‘weak’ if it included
    - <10% equivocal/ insufficient/ inconsistent 😐 SoBE graded recommendations over all included CPGs; OR
    - Equivocal/ insufficient/ inconsistent 😐 SoBE graded recommendations from included component CPGs older than five years, which may not have had the advantage of including new, more definitive literature
      - The percentage of older CPGs in the evidence dataset which met this rule was limited to 50% or less. If the percentage of older CPGs with low SoBE graded recommendations was higher than 50%, then the composite strength of the body of evidence was downgraded to **N**o **C**lear **J**udgement
  - However, if the equivocal/ inconsistent/ insufficient evidence came from one or more recent, high quality CPGs (published within the last five years), the Alper et al (2017) decision-making algorithm would be invoked and the composite recommendation strength of the composite body of evidence would be downgraded to **N**o **C**lear **J**udgement (NCJ). The reasons for downgrading the composite strength of the body of evidence would be explained in the text.

***Inconsistent, Equivocal or Insufficient (limited) Evidence***

- ***Inconsistent evidence strength and/or direction:*** When there are recommendations from three or more component CPGs with **inconsistent SoBE gradings** [☺☺☺, ☺☺, ☺, 😐, ☹, ☹☹, ☹☹☹] and / or **inconsistent evidence directions** (the evidence points in different ways), the wording states: ‘There are component recommendations from xx CPGs (*aa* high quality (HQ), *bb* moderate quality (MQ), *cc* (Poor Quality (PQ)) that provide inconsistent evidence for this question. No clear judgement can be made for or against (a care action)’. The question cannot be answered because **N**o **C**lear **J**udgement (**NCJ)** is possible
  - **NB** One approach to clarify the evidence in this scenario could be to reconsider a subset of the current evidence base, if there are sufficient recent, high quality component CPGs available to do so. CPGs published in the last five years that contribute component recommendations for this SA-cSRG question could be re-assessed as a subset for SoBE and consistency.
    - If there are three or more recent, high quality CPGs that report more consistent and/ or higher SoBE gradings, an interim composite recommendation could be proposed, and the underpinning composite SoBE determination could be referred through the relevant stronger evidence pathways (positive or negative). Caveats and limitations on the believability of the recommendation would be explained in the text
    - If there are two consistent CPGs where at least one provides a recommendation which has a higher SoBE grading, an interim composite recommendation could be proposed, and the underpinning composite SoBE determination could be graded as **I**nterim **S**uggestion (Int S). Caveats and limitations on the believability of the recommendation would be explained in the text.
- ***Equivocal / uncertain evidence:*** When there are component recommendations from three or more CPGs with consistently **equivocal/ uncertain SoBE gradings** [😐] (reflecting non-significant findings from the underpinning research), the composite SoBE wording states: ‘There are component recommendations from xx CPGs (*aa* high quality (HQ), *bb* moderate quality (MQ), *cc* (Poor Quality (PQ)) that provide an equivocal evidence base for this question. No clear judgement can be made for or against (a care action)’, and thus the question cannot be answered. Strength of the body of evidence is **NCJ** (**N**o **C**lear **J**udgement).
- ***Limited evidence from one, or two component CPGs***
  - When the SA-cSRG question is answered by only one component CPG recommendation, irrespective of its SoBE grading (☺☺☺, ☺☺, ☺, 😐, ☹, ☹☹, ☹☹☹), the composite SoBE wording is ‘There is insufficient evidence from one CPG (*aa* high quality (HQ) OR *bb* moderate quality (MQ) OR *cc* (Poor Quality (PQ))’ and thus the question cannot be answered because of **I**nsufficient evidence **(I**)
  - When the question is answered by two component CPGs:
    - Where the two component recommendations have inconsistent findings and different SoBE in their underpinning evidence base (☺☺☺, ☺☺, ☺, 😐, ☹, ☹☹, ☹☹☹), the composite SoBE strength of the body of evidence wording is ‘There is insufficient/ inconsistent evidence from two inconsistent, different strength evidence base CPGs (*aa* high quality (HQ), *bb* moderate quality (MQ) OR *cc* (Poor Quality (PQ))’, thus the question cannot be answered because **NCJ** (**N**o **C**lear **J**udgement) is possible.
      - If one of these CPGs is current and high quality, this component recommendation could be referred through the pathway for one CPG, and an **I**nsufficient evidence statement (I) could be made with an appropriate justification
    - Where both component CPGs are current, have consistent direction component recommendations, and at least one component recommendation has a high SoBE, the strength of the body of evidence wording is ‘Interim support is provided on the basis of two current, consistent CPGs (aa HQ, bb MQ)’ (graded Int S (**I**nterim **S**upport)).

For all examples of Inconsistent, Equivocal or Insufficient (limited) Evidence, the composite SoBE determinations (No Clear Judgement, Insufficient evidence, Interim Support) could provide impetus for research to provide a stronger evidence base for the question.

***Negative evidence (Against)***

- ***Strong Consistent Evidence Against (a care action):*** When the composite recommendation is underpinned by three or more component CPG recommendations that are generally supported by **high SoBE gradings,** which provide **negative consistent recommendations (against** a care action) [☹☹☹], the composite SoBE wording states: ‘There are Consistent and Strong Recommendations from *xx* CPGs (*aa* high quality (HQ), *bb* moderate quality (MQ), *cc* (Poor Quality (PQ))’ **Against** (a particular care action). This composite recommendation strength of the body of evidence has three ‘crosses’ ✖✖✖.
  - The evidence base could still be called ‘strong’ if it included
    - <10% individual moderate [☹☹] SoBE graded recommendations against (a particular care action) considering all included CPGs; OR
    - moderate [☹☹] SoBE graded recommendations against (a care action) from composite CPGs older than five years, which may not have had the benefit of including new more definitive literature.
      - The percentage of older CPGs in the evidence dataset which met this rule was limited to 50%. If the percentage of older CPGs with moderate [☹☹] composite SoBE graded recommendations was higher than 50%, the composite SoBE was downgraded to ✖✖.
  - However, if the moderate SoBE gradings [☹☹] underpin recommendations extracted from one or more recent, high quality component CPGs, the Alper et al (2017) decision-making algorithm would be invoked, and the composite recommendation SoBE would be downgraded to ✖✖. The reasons for this decision would be explained in the text.
- ***Moderate Consistent Evidence Against (a care action):*** When the composite recommendation is underpinned by three or more CPG recommendations with **moderate rSoBE gradings,** which provide **negative consistent recommendations** **against** (a care action) (☹☹) the composite SoBE wording is ‘There were Consistent Suggestions from *xx* CPGs (*aa* high quality (HQ), *bb* moderate quality (MQ), *cc* (Poor Quality (PQ)) **Against** (a particular care action)**’.**  The composite recommendation is given two ‘crosses’ ✖ ✖
  - The evidence base could still be called ‘moderate’ if it included
    - <10% low SoBE recommendations [☹] against (a particular care action) over all included CPGs for that question; OR
    - Low SoBE recommendations [☹] against (a care action) from CPGs older than five years, which may not have had the advantage of including new, more definitive literature
      - The percentage of older CPGs in the evidence dataset which met this rule was limited to 50%. If the percentage of older CPGs with low [☹] SoBE recommendations was higher than 50%, the strength of the body of evidence was downgraded to ✖.
  - However, if the low SoBE grading (☹) underpins recommendations extracted from one or more recent, high quality component CPGs, the Alper et al (2017) decision-making algorithm would be invoked and the composite SoBE would be downgraded to ✖. The reasons underpinning this decision would be explained in the text.
- ***Weak Consistent Evidence Against*** **(a care action)**: When the composite recommendation is underpinned by three or more CPG recommendations with **low SoBE gradings** which provided **consistent negative recommendations against** (a particular care action) [☹], the composite SoBE wording is ‘There was Weak Support from *xx* CPGs (*aa* high quality (HQ), *bb* moderate quality (MQ), *cc* (Poor Quality (PQ)) **Against** (the care action)’. The composite recommendation is given one ‘cross’ ✖.
  - The evidence base could still be called ‘weak’ if it included
    - - <10% equivocal / inconsistent / insufficient evidence recommendations [😐] regarding a care action, over all included CPGs for that question; OR
      - Equivocal/ insufficient/ inconsistent (😐) SoBE recommendations from CPGs older than five years, which may not have had the advantage of considering new more definitive literature
        - The percentage of older CPGs in the evidence dataset which met this rule was limited to 50%. If the percentage of older CPGs with equivocal [😐] SoBE graded recommendations was higher than 50%, the composite SoBE was downgraded to NCJ.
  - However, if the equivocal/ insufficient / inconsistent evidence (😐) came from more recent, high quality CPGs (within the last five years), the Alper et al (2017) decision-making algorithm would be invoked and the composite evidence body strength would be downgraded to **N**o **C**lear **J**udgement (NCJ). The reasons for this decision would be explained in the text.

**APPENDIX 5.** AGREE II domain scores expressed as % of possible total domain score

| **Guidance documents** | **Domain 1** | **Domain 2** | **Domain 3** | **Domain 4** | **Domain 5** | **Domain 6** |
| --- | --- | --- | --- | --- | --- | --- |
| ACSQH [40] | 88.9 | 22.2 | 20.8 | 30.6 | 4.2 | 4.2 |
| AHA/ASA [37] | 88.9 | 63.9 | 84.4 | 83.3 | 37.5 | 25.0 |
| AHRQ [39] | 94.4 | 52.8 | 97.9 | 72.2 | 43.8 | 25.0 |
| AHRQ_OT [38] | 100.0 | 63.9 | 97.9 | 72.2 | 14.6 | 50.0 |
| Aust Stroke Foundation [41] | 100.0 | 16.7 | 97.9 | 69.4 | 10.4 | 25.0 |
| Can Stroke Guidelines [42] | 100.0 | 94.4 | 99.0 | 94.4 | 37.5 | 70.8 |
| VA/DoD [43] | 100.0 | 61.1 | 99.0 | 94.4 | 37.5 | 0.0 |
| Malaysia [44] | 100.0 | 61.1 | 97.9 | 80.6 | 45.8 | 87.5 |
| NICE [46] | 94.4 | 55.6 | 100.0 | 83.3 | 33.3 | 50.0 |
| NSW ACI [47] | 88.9 | 33.3 | 100.0 | 63.9 | 16.7 | 41.7 |
| NZGG [45] | 77.8 | 33.3 | 89.6 | 69.4 | 89.6 | 50.0 |
| RCP [48] | 100.0 | 100.0 | 100.0 | 88.9 | 77.1 | 50.0 |
| Sth Aust Stoke Clin Network [49] | 94.4 | 61.1 | 100.0 | 86.1 | 47.9 | 50.0 |
| Sth Afr Stroke Guidelines [52] | 80.6 | 44.4 | 28.1 | 86.1 | 35.4 | 100.0 |
| SIGN Dysphasia [50] | 100.0 | 50.0 | 100.0 | 77.8 | 60.4 | 75.0 |
| SIGN rehab [51] | 97.2 | 83.3 | 100.0 | 91.7 | 16.7 | 75.0 |

**APPENDIX 6.** Example of the process used to develop the wording for composite recommendations

**Step 1:** For this example, three project questions were considered together as the individual guidance documents often presented the evidence together.

2. What is the optimal time for referral to rehabilitation since admission to hospital?

3. What is the optimal time for commencement of rehabilitation since suffering a stroke?

4. What is the optimal time for referral to rehab since admission to hospital?

**Step 2.** Recommendations and SBoE extracted from each relevant guidance document

| **Guidance document** | **Evidence statement** | **SBoE** |
| --- | --- | --- |
| ACSQHC [40] | A patient’s rehabilitation needs and goals are assessed by staff trained in rehabilitation within 24–48 hours of admission to the stroke unit. Rehabilitation is started as soon as possible, depending on the patient’s clinical condition and their preferences. | cites other CPGs (no SoBE) |
| AHA/ASA [37] | It is recommended that stroke patients who are candidates for postacute rehabilitation receive organized, coordinated, interprofessional care whilst in acute settings.  There are benefits to starting rehabilitation as soon as the patient is ready and can tolerate it   - An inpatient rehabilitation facility (IRF) should provide at least 3 hours of rehabilitation therapy (defined as PT, OT, and SLT) per day for at least 5 d/wk. - Specialist Nursing Facilities (SNFs) are required to have rehabilitation nursing on site for a minimum of 8 h/d | A  A  B  B |

| Aust Stroke Foundation [41] | Every stroke patient should have their rehabilitation needs assessed in the first week of stroke by members of the multidisciplinary team | A |
| --- | --- | --- |

| Can Stroke Guidelines [42] | Initial screening and assessment should be commenced within 48 hours of admission by rehabilitation professionals in direct contact with the patient.  Initial assessment would include: an evaluation of patient function, safety, physical readiness, and ability to learn and participate in rehabilitation therapies  Patients should receive a recommended three hours per day of direct task-specific therapy, five days a week, delivered by the inter-professional stroke team  The more therapy results in better outcomes  Patients should receive rehabilitation therapies of appropriate intensity and duration, individually designed to meet their needs for optimal recovery and tolerance levels | C  C  C  A  A |
| --- | --- | --- |
| AHQR [39] | A comprehensive assessment of a person with stroke should take into account:   - Their previous functional abilities - Impairment of psychological functioning (cognitive, emotional, and communication) - Impairment of body functions, including pain - Activity limitations and participation restrictions - Environmental factors (social, physical, and cultural)   Information collected routinely from people with stroke using valid, reliable, and responsive tools Information collected from people with stroke using valid, reliable, and responsive tools should be fed back to the multidisciplinary team regularly | A |
| VA / DoD guideline [43] | Early assessment and intervention is critical to optimize rehabilitation.  The primary goal of rehabilitation is to prevent complications, minimize impairments, and maximize function  Secondary prevention is fundamental to preventing stroke recurrence  Evidence-based interventions should be based on functional goals.  Patient and family education improves informed decision-making, social adjustment, and maintenance of rehabilitation gains.  It is strongly recommended that rehabilitation therapy should start as early as possible, once medical stability is reached.  It is recommended that the patient receive as much therapy as “needed” and tolerated to adapt, recover, and/or reestablish the premorbid or optimal level of functional independence. | A  A  A  A  B  A  GPP |
| NSW ACI [47] | Rehabilitation should be commenced as soon as possible on the acute stroke unit. Acute stroke service providers require effective links with stroke rehabilitation service providers to optimize outcomes for people with stroke [3,4] | A |
| RCP [48] | Patients with difficulty moving early after stroke, and who are medically stable, should be offered frequent, short daily mobilization (sitting out of bed, standing or walking) by appropriately trained staff with access to appropriate equipment, typically beginning between 24-48 hours of stroke onset. Mobilisation within 24 hours of onset should only be provided for patients who require little or no assistance to mobilise. | A |
| SIGN [52] | Stroke patients should be mobilised as early as possible after stroke.  A full understanding of the patient’s cognitive strengths and weaknesses should be an integral part of the rehabilitation plan | B  GPP |
| South Africa [53] | Early initiation of rehabilitation is recommended  Early discharge from stroke unit care is possible in medically stable patients with mild or moderate impairment, providing that rehabilitation is delivered in the community by a multidisciplinary team with stroke expertise | C  A |

**Step 3.** Identify relevant words and phrases from individual recommendations

***Key:*** *Grey highlights relate to time, yellow highlights relate to safety*

| Q2. What is the optimal time for referral to rehabilitation since admission to hospital? | Rehabilitation commenced within 24–48 hours of admission [40]  Rehabilitation commenced as soon as the patient is ready and can tolerate it [37]  Rehabilitation needs assessed in the first week of stroke [41]  Rehabilitation is started as soon as possible [40]  Initial screening and assessment should be commenced within 48 hours of admission [42]  Early assessment and intervention is critical to optimize rehabilitation [43]  Rehabilitation therapy should start as early as possible, once medical stability is reached [43].  Rehabilitation should be commenced as soon as possible[47]  Typically beginning between 24-48 hours of stroke onset [48]  Stroke patients should be mobilised as early as possible after stroke [52]  Early initiation of rehabilitation is recommended [53] . |
| --- | --- |
| Q3. What is the optimal time for commencement of rehabilitation since suffering a stroke |  |
| Q4. What is the optimal time for referral to rehab since admission to hospital? |  |

**Step 4.** Consider the uniformity of wording and intent in extracted text from the recommendations [24]. For these three exemplar questions, there is consistent wording and intent, as outlined by the shading in Step 3, for two themes (medical stability and early commencement of rehabilitation).

**Step 5**. Formulate overall recommendations to answer project questions:

- Refer to AH rehabilitation immediately the patient is medically stable
- Conduct comprehensive assessments within 48 hours of receiving referral to rehabilitation
- Commence multidisciplinary rehabilitation within two days of referral

**Step 6.** Present and defend the recommendation amalgamation process to the project team, and make adjustments to wording as required.

**Step 7.** ‘Back check’ the composite recommendation against the recommendations from the individual guidance documents to ensure that no nuances have been lost in the process.

**APPENDIX 7.** Composite recommendations and OSoBE gradings

**Key:** Bolded text and red OSoBE gradings highlighted the overarching recommendations, and the ordinary text and black OSoBE gradings reflect sub-recommendations.

**1. ORGANISE FOR BEST PRACTICE REHABILITATION**

| ***1.1*** | ***Establish best practice multidisciplinary AH stroke rehabilitation teams at all points of entry to the healthcare system*** | *✔✔✔* |
| --- | --- | --- |
| *✔✔✔* | People who suffer from stroke should be seen by a multidisciplinary/ inter-professional/ interdisciplinary stroke team for medical and rehabilitation assessment and management. The team consists of doctors, nurses, physiotherapists, occupational therapists, speech language therapists, social workers, dieticians, clinical neuropsychologists/clinical psychologists. | |
| *✔✔* | All members of the multiD team should have specialized training in stroke care and recovery. | |
| ***1.2*** | ***Establish clear models of care relevant to different points of entry to the***  ***healthcare system in the South African system I*** | |
| ✔✔✔ | All patients who suffer from stroke should have access to specialist stroke service units with multidisciplinary team as early as the hyper-acute – acute stages of stroke and up to discharge. | |
| ✔✔✔ | Stroke units should incorporate rehabilitation services | |
| ✔✔✔ | Rehabilitation processes should commence in the acute setting as soon as the person with stroke is medically safe and/or able to participate. | |
| ✔✔ | A standard set of assessment tools should be used to assess rehabilitation needs throughout the patient journey. These should be valid, sensitivity to detect change, simple to use, and if required, apply standard protocols to assist more complex assessment. | |

**2. OPERATIONALISE STRATEGIES FOR BEST PRACTICE COMMUNICATION, RISK MINIMIZATION AND PLANNING THROUGHOUT THE PATIENT JOURNEY**

| ***2.1*** | ***Minimise risks of adverse events after stroke*** | *✔✔* |
| --- | --- | --- |
| ✔✔ | All patients who suffer from stroke to have their swallowing capacity screened by a trained health professional before taking any food, drink and oral medication | |
| ✔✔ | The water swallow test should be performed at the bedside, as a useful screening tool for dysphagia. *The* *Toronto Bedside Swallowing Screening test, an evidence-based tool for swallow assessment, has been evaluated successfully for interrater reliability and predictive validity (Martino et al 2009).* | |
| ✔✔ | A standardised clinical bedside assessment (CBA) should be applied by a professional skilled in the management of dysphagia (currently speech and language therapists) | |
| ✔✔ | A speech and language therapist (SLT) should perform the swallow screen test. If a SLT is not available, the test should be undertaken by another appropriately-trained professional | |
| ✔✔✔ | Videofluoroscopic swallow studies (VSS, VFSS,) or fiberoptic endoscopic examination of swallowing (FEES) should be performed on all patients considered at risk for pharyngeal dysphagia or poor airway protection, based on results from the bedside swallowing assessment. | |
| ✔✔ | Education should be made available to all healthcare providers about adverse events following stroke | |
| ✔✔ | This should include information on risks for stroke, stroke prevention, potential for re-stroke, the importance of swallow screen to prevent pneumonia, skin care and pressure sore prevention, prevention of falls, management of depression, prevention of shoulder subluxation. | |
|  | There is no evidence about the best way that this information should be provided. | |
| ✔✔ | All patients who suffer from stroke should be mobilized as early as possible, to lessen likelihood of complications such as pneumonia, DVT, PE, and pressure sores. | |
| ✔✔ | Patients with mild and moderate stroke should be provided with frequent, short sessions of out of bed activity. | |
| **NCJ** | No clear judgement can be made on the optimal timing for mobilization. | |
| ✖✖✖ | **R**outine use of splints or prolonged positioning of upper or lower limb muscles in a lengthened position (stretch) for patients with stroke who are at risk of developing contracture is contraindicated | |
| ✔✔✔  ✔✔✔ | All stroke survivors should undergo pressure care risk assessment and regular evaluation, completed by trained personnel.   - Patients assessed as high risk should be provided with appropriate pressure relieving aides and strategies, including a pressure relieving mattress as an alternative to a standard hospital mattress or appropriate seating systems | |
| ✔✔✔  ✔✔✔ | All stroke survivors should undergo falls risk assessment using a validated tool on admission to hospital.   - An interdisciplinary management plan should be initiated for all those identified as at risk of falls. | |
| ***2.2*** | ***Ensure that patient and family is engaged in relevant discussions and decisions*** *✔✔*  ***throughout the patient journey*** | |
| ✔✔ | Patients, family and carers should be involved in planning rehabilitation goals and management, problem solving and decision-making, and be given formal and informal education on stroke rehabilitation | |
| ✔✔ | Patient and family education, and family support, should commence once the patient’s condition is stabilised, and continue throughout the rehabilitation process | |
| ***2.3*** | ***Communicate effectively with others about rehabilitation plans and progress INT S*** | |
| INT S | The timing of communication and meetings between the patients, family members and health professionals is not clear. | |
| INT S | Communication should ideally be commenced, and led by, one nominated key worker identified by the multidisciplinary team | |
| INT S | It is not clear whether the team social worker (or any other health professional) is the best person to lead this communication. | |
| INT S | Individually tailored information, education and training may be of benefit. | |
| ✔✔ | Communication should include:   - written information about stroke, the rehabilitation process, referrals, appointments, GP discharge summary individualized for the needs of the patients and carers - a mixture of education and counselling techniques - behavior change for long term prevention | |
| ✔✔ | Communication should be tailored to meet the needs of the patients/family using relevant language and communication format, with opportunities for follow up, clarification and reinforcement | |
| NCJ | It is not clear whether contact with, and education by, trained staff should be offered to all people with stroke, and family/carers after discharge | |
| INT S | Communication between the health professionals (medical and therapists) could occur via multidisciplinary meetings and case conferences, as well as in liaision with other health professionals through networks. | |
| INT S | Communication between health professionals, patients and relatives could occur regularly, formally and informally | |
| I | There is insufficient evidence regarding alternative methods of communication and support (eg telephone visits, telehealth, or Web-based support), particularly for patients in rural settings | |
|  | There is no evidence regarding communication platforms for therapist/employers | |
| INT S | Psychological assessment and intervention could be undertaken for patients with reduced motivation, self-esteem and/or self-efficacy | |
| INT S | The use of psychological principles from motivational interviewing and problem solving should be incorporated into education programs for people who have suffered a stroke | |
| I | There is insufficient evidence to suggest that offering routine psychological therapies in one-to-one format following a stroke is not recommended to prevent post-stroke depression | |
| I | There is insufficient evidence that each multidisciplinary stroke team should have access to a clinical psychologist (SIGN 2010) | |
| I | There is insufficient evidence that the criteria for admission to any rehabilitation setting should be standardized and communicated to all referring centers and services | |
| ***2.4*** | ***Establish active plans early within the patient journey to reintegrate stroke***  ***patients into their community ✔✔✔*** | |
| ✔✔✔ | Patients should be given support to re-integrate in the community. Patients should be given opportunities to participate in organized community-based and coordinated inter-professional rehabilitation care, social activities, provided with information and referral to statutory and non-statutory community organisations that can support the person in social participation. | |
| **I** | There is insufficient evidence that people with stroke whose social behaviour is causing distress to themselves or others should be assessed by an appropriately trained healthcare professional to determine the underlying cause, and advise on management. Following the assessment:   - the nature of the problem and its cause should be explained to family/carers, other people in social contact and the rehabilitation team; - the person should be helped to learn the best way to interact without causing distress; - those involved in social interactions should be trained in how to respond to inappropriate or distressing behaviour;   psychosocial management approaches should be considered | |
| **I** | To ensure long-term maintenance of health benefits, a planned transition could be implemented from structured aerobic exercise to more self-directed physical activity at home or in the community | |
| ***2.5*** | ***Support self-efficacy principles and training for patients and family NCJ*** | |
| Int S | Patients’ self-management could be assessed early in the hospital admission. | |
| Int S | Patients could be trained for self-management to do compensatory techniques, to be able to overcome barriers to engagement in active activities and to engage in social and leisure activities | |
| **I** | People who have had a stroke who are cognitively able should be made aware of the availability of generic self-management programs before discharge from hospital and be supported to access such programs once they have returned to the community. Stroke-specific programs for self-management should be provided for those who require more specialised programs | |

**3. ADMIT TO ACUTE HOSPITAL**

| ***3.1*** | ***Reduce delay in admission to a medical facility for people suffering stroke ✔✔*** |
| --- | --- |

| ✔✔ | Delays should be reduced so that people suspected of suffering a stroke receive the medical treatment they require in the shortest time possible |
| --- | --- |
| ✔✔ | It is importance that the public, and health professionals are educated in the use of the *F.A.S.T.* assessment instrument to recognize stroke symptoms, and to minimize delays for patients in reaching medical care quickly, and in recognizing subsequent strokes in stoke survivors. |

**4. REFER TO INPATIENT REHABILITATION**

| ***4.1*** | ***Refer to AH rehabilitation immediately the patient is medically stable*** | ***✔✔✔*** |
| --- | --- | --- |

| ✔✔✔ | Patients suffering from stroke should be referred to a multidisciplinary stroke unit as soon as he/she is deemed to be medically stable and able to participate safely in rehabilitation |
| --- | --- |
| ✔✔✔ | AH staffing in the multidisciplinary acute stroke unit should include physiotherapy, occupational therapy, speech-language pathology, dietetics, clinical psychology and social work |
| ✔✔ | Patients with stroke and their families should be integral members of the multidisciplinary stroke team |
| ✔✔ | Patients’ rehabilitation progress should be regularly assessed, documented centrally in a manner accessible to all, and matched with patient and family goals |
| ✔✔ | The stroke team should determine when patients are ready (and safe) to commence rehabilitation |
| ✔✔ | Formal and informal team meetings should occur regularly |
| ✔✔ | Rehabilitation decisions should be clearly documented |
| ✔✔ | Documentation should be in a central place accessible to all rehabilitation team members (i.e. in written or electronic patient case notes) |
| NCJ | No clear judgement can be made as to whether one Key Worker (or multiple Key Workers with specific roles) should be assigned to each patient with stroke, and be their point of liaison throughout the hospital stay. |

**5. ACTION INPATIENT REHABILITATION**

| ***5.1*** | ***Conduct comprehensive assessments within 48 hours of receiving referral to rehabilitation*** | ***✔✔*** |
| --- | --- | --- |

| ✔✔ | Stroke patients should be screened as early as possible after admission to hospital for rehabilitation potential |
| --- | --- |
| ✔✔✔ | Stroke patients should be screened as early as possible after admission to hospital for risks of adverse events |
| ✔✔✔ | Stroke patients’ specific rehabilitation needs should be comprehensively assessed within 48 hours of admission to acute hospital, or when the patient is considered to be medically stable (whichever comes first) |
| ✔✔ | Rehabilitation should be provided within stroke patients’ level of tolerance |
| ✔✔ | Comprehensive assessment of rehabilitation needs should include:   - Previous functional abilities - Impairment of psychological functioning (cognitive, emotional, and communication) - Impairment of body functions, including pain - Activity limitations and participation restrictions   Environmental factors (social, physical, and cultural) |
| ✔✔✔ | Patients should commence rehabilitation when they are considered to be medically stable, and when their risks of complications or adverse events are managed |
| ✔✔✔ | Rehabilitation plans should be designed to meet patient goals and needs for recovery, within their level of tolerance |
| ✔✔ | Stroke patients should be screened immediately following admission for:   - orientation - positioning, moving and handling - swallowing - transfers (for example, from bed to chair) - pressure area risk - continence - communication, including the ability to understand and follow instructions and to convey needs and wishes - nutritional status and hydration |
| ✔✔ | A standard assessment process should follow within 24-48 hours of admission to hospital, once the patient is able to tolerate it |
| I | There is insufficient evidence regarding the components of a standard comprehensive assessment process, including assessment items, frequency of assessment and efficient documentation available to all. |
| ✔✔ | *The ART assessment instrument is a comprehensive international tool which enables comparisons between sites* |
| ✔✔ | Routine use of standard outcome measures should occur to collect information from patients in the functional domains affected by the stroke |
| ✔✔ | Measurement of changes over time in repeated administrations of standard outcome measures should be used to underpin decisions regarding ongoing rehabilitation |
| ✔✔ | The more therapy is provided, the better the outcome |

| ***5.2*** | ***Commence multidisciplinary rehabilitation within two days of referral*** *✔✔✔* |
| --- | --- |

| ✔✔✔ | Rehabilitation should commence as early as possible (suggested as within two days of medical stability being reached, and when risk of adverse events and complications are minimised) |
| --- | --- |
| ✔✔✔ | Rehabilitation activities and intensity should be provided within patient tolerance, needs and choice |
| ✔✔✔ | Physiotherapists, occupational therapists, speech and language therapists and dieticians bring specific competencies and skills to patient assessment and rehabilitation planning. They operate most effectively when sharing the assessment and rehabilitation tasks, and communicating findings verbally and in written form in patient notes, as members of the multidisciplinary team. |
| ✔✔ | Task sharing in assessment and rehabilitation leads to more comprehensive assessment, and integrated rehabilitation planning |
| ✔✔ | Patient-specific rehabilitation management strategies should be developed on the basis of findings from comprehensive patient deficit and need assessments. |
| ✔✔  ✔✔✔  ✔✔✔  NCJ  NCJ  NCJ  ✔✔✔  ✔✔  ✔✔✔  ✔✔  NCJ | *In summary:*   - Skin condition should be closely monitored to prevent breakdown - Subluxation of hemiplegic shoulder should be prevented, and if it occurs, minimize pain and dysfunction   Falls should be prevented by improving balance  No clear guidance can be provided for the effectiveness of electrotherapy or medication (eg amitriptyline) for central pain.  No clear guidance can be provided for the use of strategy or gesture training, or task practice for patients with apraxia.  No clear guidance can be provided regarding the use of exercise for cognitive deficits.  Partner training should be included for intensive treatment for communication disorders  Urinary and fecal incontinence should be reduced using individually-tailored approaches.  To improve lower limb mobility:   - intensive, repetitive mobility-task training exercises, repetitive walking, ankle orthotics, circuit training and group therapy, robot assisted movement training should be used - electrotherapy, rhythmic auditory cueing, biofeedback, virtual reality, water-based exercise, mechanically-assisted walking can be suggested - No clear guidance can be provided for use of acupuncture |
| ✖✖✖  ✖✖  ✔✔  ✔✔  ✔✔  ✔✔ | To improve upper limb mobility and function:   - Do not use resting splints to reduce spasticity in wrist or fingers - Do not use splints, repetitive task training or intensive therapy - Constraint-induced movement therapy should be considered for individuals with intact balance, cognition and at least 10deg finger extension - The use of mental practice should be considered as an adjunct to normal practice to improve upper limb function after stroke - The use of robotic / electromechanical devices in selected patients should be considered, where equipment and expertise is available   Gait should be retrained using a range of interventions. |
| ✔✔ | Individually tailored strategies should be considered for speech disorders, including behavioural strategies, and physiological supports |

| ***5.3*** | ***Set achievable rehabilitation goals*** | ***✔*** |
| --- | --- | --- |

| I | *Rehabilitation goals should be tailored to patients’ capacity to improve, tolerance to rehabilitation, general health, family and social circumstances, and planned discharge destination* |
| --- | --- |
| Int S | Standard outcome measures could be collected over-time data on each element of patient rehabilitation. |
| INT S | Outcome measures should be sensitive over time, and to have published minimum clinical significant differences with which to compare patient progress |

| ***5.4*** | ***Use best practice recording methods for assessment, treatment and goal setting ✔✔*** |
| --- | --- |

| ✔✔✔ | Standard, published assessment and outcome measures should be used regularly throughout the patient’s hospital stay. |
| --- | --- |
| ✔✔ | Treatment decisions should be clearly documented, including intervention choice (and reason for choice), frequency of intervention and response to it |
| ✔✔ | Progression of rehabilitation programs should be documented, including reason for progression, and patient responses |
| ✔✔✔ | All documentation should be recorded in legible format in a central place accessible to the stroke team |
| ✔✔ | Progress reports on interventions and outcomes should be communicated regularly within the stroke care team, and to the patient and family |

| ***5.5*** | ***Record outcomes effectively along the patient journey*** | ***✔✔*** |
| --- | --- | --- |

|  | See above recommendations |
| --- | --- |

| ***5.6*** | ***Provide appropriate aids and assistive technology in a timely manner*** | *✔✔* |
| --- | --- | --- |

| ✔✔ | Walking aids should be considered only after a full assessment of the potential benefits and harms of the walking aid in relation to the individual patient’s stage of recovery and presentation |
| --- | --- |
| ✔✔ | Ambulatory assistive devices should be used where appropriate, to optimize gait and balance impairments, and improve mobility efficiency and safety |
| NCJ | No clear guidance can be provided regarding whether AFOs should be used for ankle instability or dorsiflexor weakness. |
| NCJ | No clear guidance can be provided regarding whether wheelchairs should be used for non-ambulatory individuals or those with limited walking ability. |
| ✔✔ | Adaptive and assistive devices should be used for safety and function, if other methods of performing the task/activity are not available or cannot be learned or if the patient’s safety is a concern. |

**6. DISCHARGE FROM INPATIENT REHABILITATION**

| ***6.1*** | ***Establish discharge plans early in the hospital stay*** | ***✔✔✔*** |
| --- | --- | --- |

| *✔✔✔* | Discharge planning (DCP) for patients with stroke should commence from day 1 of admission to the acute hospital |
| --- | --- |
| *✔✔✔* | DCP should include all members of the multiD team, and the patient and family |
| *✔✔✔* | DCP should consider patient and family circumstances (financial, social, housing, employment, family responsibilities) as well as patient goals, and family capacity to assist the patient to meet these goals |
| *✔✔✔* | DCP should include patients’ capacity to be rehabilitated |
| *✔✔* | DCPs should be revised regularly throughout the patient journey (inpatient and after discharge to community care) |
| *✔✔* | Revision of DCPs should articulate with re-assessments of patient progress and goals |
| *✔✔* | Every rehabilitation professional should take responsibility for planning and monitoring continuation of care |
| *✔✔✔* | OT home visits should be conducted prior to the patient returning home |
| ✔✔✔ | Information about patient progress should be recorded formally in patient notes and shared at discharge planning meetings |
| ✔✔ | One member of the multiD team should take overall carriage of DCP to ensure continuity |
| **NCJ** | There is no clear guidance whether social workers, dedicated discharge planners or occupational therapists are best placed to take overall carriage of DCP |
| ✔✔ | The family should be involved in DCP as early as possible in the patient admission to acute hospital |

| ***6.2*** | ***Ensure best practice, timely referral to community care, and other mainstream health professionals*** | *✔✔* |
| --- | --- | --- |

| ✔✔ | DCP should incorporate transition from acute hospital to community rehabilitation as an integral part of the patient journey. |
| --- | --- |
| ✔✔ | DCP should be communicated early with community care providers, to ensure that appropriate care and supports are available for patients as soon as they are discharged from acute hospitals |

**7. LONGER TERM COMMUNITY-BASED REHABILITATION**

| ***7.1*** | ***Regularly evaluate and record rehabilitation progress*** | *✔✔* |
| --- | --- | --- |
| ✔✔ | Rehabilitation progress should be regularly evaluated by the multidisciplinary team, and recorded in a standard manner in patient notes | |
| ✔✔ | Achievable and agreed rehabilitation goals should be set and recorded formally in patient notes. Goals should be used for re-evaluation and regularly reassessment | |
| ✔✔ | Essential items to record when assessing and treating a stroke patient should include, but are not limited to:   - Diagnosis and health status - Mental capacity - Functional abilities - Transfers and mobility - Care needs for washing, dressing, - toileting and feeding - Psychological and emotional needs - Medication needs - Social circumstances - Management of risk including the needs of vulnerable adults - Ongoing goals - Ways of accessing rehabilitation services | |
| ✔✔ | The next best level of care should be considered after discharge from hospital. If the patient is discharged to home, ongoing rehabilitation should be available, and could be provided in home, at local community centres, outpatient clinics, or rehabilitation centres. If the patient is discharged to residential care, ongoing rehabilitation may be provided ‘in house’ or in a community centre. | |
| ✔✔ | Longer term care for stroke survivors should reflect their goals and circumstances. | |
| ✔✔ | There is no ‘one size fits all’ guidance for long term stroke rehabilitation. | |
| ✔✔ | Long term rehabilitation should be patient and family/ carer-centred. These could include interventions to maintain and improve mobility; education on sexuality, return to work and driving; interventions for mental health and community re-engagement. | |
|  | There is no evidence from these CPGs regarding what interventions should be provided in different settings (rehab facility; Community Health Center (CHC); long term home care; home or community) | |
| ***7.2*** | ***Consider the role of traditional healers in local contexts*** | *I* |
| I | There is insufficient evidence to answer this question | |
| ***7.3*** | ***End active community rehabilitation when there is no further benefit, but***  ***monitor as needed NCJ*** | |
| NCJ | There is no clear guidance whether patients should be discharged from outpatient care when no more improvement is being reported, and/ or when patients are managing well in the community. No clear judgement can be made. | |
| NCJ | There is no clear guidance regarding timing of discharge from rehabilitation. It could be considered at least six months after discharge from hospital, but could occur up to 12 months after discharge from hospital. | |

**APPENDIX 8**. RIGHT reporting statement

| 1a | In the title line 1 |
| --- | --- |
| 1b | Line 143 |
| 1c | Lines 126-128 |
| 2 | Lines 43- 48 |
| 3 | Lines 477-472 |
| 4 | Line 15 |
| 5 | Lines 60 -125 |
| 6 | Lines 126- 128 |
| 7a | Directly Allied Health Providers (Lines 86-90); indirectly stroke survivors (Lines 111-115) |
| 7b | none |
| 8a | Lines 86-90, 169-170 |
| 8b | Line 145 |
| 9a | Lines 146-142 |
| 9b | As authors and acknowledgements |
| 10a | See Appendix 3 |
| 10b | Not relevant |
| 11a | Existing guidance documents (lines 187-189) |
| 11b | References 37-52, Lines 190-205 |
| 12 | Appendix 4 and Figure 2 |
| 13a | Appendix 7 |
| 13b | Not relevant |
| 13c | Appendix 7 |
| 14a | Consumer representative was a member of the project team |
| 14b | Resource issues were implicit in question formulation, Lines 209-241 |
| 14c | Questions were formulated on the basis of ‘average’ stroke patient journeys in the public sector, and issues in providing allied health care throughout these journeys (equity, access, timeliness) |
| 15 | Lines Appendix 6 outlines the decision-making process on recommendations, Lines 278-300 |
| 16 | Recommendations were discussed by the large project team during guideline writing. Wider public consultation will occur when the SA guideline is endorsed by the project team |
| 17 | Internal quality assurance processes were implicit in the internal discussions about draft recommendations and the processes of arriving at them Lines 297-300; Lines 319-321 |
| 18a | Lines 133-135; Lines 140-142 |
| 18b | Lines 134-135 |
| 19a | There were no conflicts, rather robust discussions about wording of recommendations |
| 19b | Discussions re wording of recommendations were managed by the researchers, and were integral to the guideline writing process and endorsement stage |
| 20 | From the corresponding author, Stellenbosch University Dept of Physiotherapy |
| 21 | Questions which could not be answered from the included guidance documents (for which there is no clear evidence) are:   - No clear judgement can be made on the optimal timing for mobilization. - It is not clear whether contact with, and education by, trained staff should be offered to all people with stroke, and family/carers after discharge - No clear judgement can be made as to whether one Key Worker (or multiple Key Workers with specific roles) should be assigned to each patient with stroke, and be their point of liaison throughout the hospital stay. - No clear guidance can be provided for the effectiveness of electrotherapy or medication (eg amitriptyline) for central pain. - No clear guidance can be provided for the use of strategy or gesture training, or task practice for patients with apraxia. - No clear guidance can be provided regarding the use of exercise for cognitive deficits. - No clear guidance can be provided for use of acupuncture - No clear guidance can be provided regarding whether AFOs should be used for ankle instability or dorsiflexor weakness. - No clear guidance can be provided regarding whether wheelchairs should be used for non-ambulatory individuals or those with limited walking ability. - There is no clear guidance whether social workers, dedicated discharge planners or occupational therapists are best placed to take overall carriage of DCP - There is no clear guidance whether patients should be discharged from outpatient care when no more improvement is being reported, and/ or when patients are managing well in the community. No clear judgement can be made. - There is no clear guidance regarding timing of discharge from rehabilitation. It could be considered at least six months after discharge from hospital, but could occur up to 12 months after discharge from hospital.   Targeted research to address these questions should be conducted. |
| 22 | The limitations were that a new process was developed and tested on one set of questions (allied health stroke rehabilitation). This condition provided a vehicle. The project team was learning as it went and errors and mis-interpretations were sure to have been made, particularly in the formulation of composite recommendations from the included guidance documents. The process of combining evidence sources into one composite set of recommendations needs to be tested by other teams for other conditions |
